# Early micro‐ and macrostructure of sensorimotor tracts and development of cerebral palsy in high risk infants

**DOI:** 10.1002/hbm.25579

**Published:** 2021-07-29

**Authors:** Rahul Chandwani, Julia E. Kline, Karen Harpster, Jean Tkach, Nehal A. Parikh, The Cincinnati Infant Neurodevelopment Early Prediction Study (CINEPS) Group

**Affiliations:** ^1^ Perinatal Institute Cincinnati Children's Hospital Medical Center Cincinnati Ohio USA; ^2^ Division of Occupational Therapy and Physical Therapy Cincinnati Children's Hospital Medical Center Cincinnati Ohio USA; ^3^ Department of Rehabilitation, Exercise and Nutrition Sciences University of Cincinnati College of Allied Health Sciences Cincinnati Ohio USA; ^4^ Department of Radiology Cincinnati Children's Hospital Medical Center Cincinnati Ohio USA; ^5^ Imaging Research Center, Department of Radiology Cincinnati Children's Hospital Medical Center Cincinnati Ohio USA; ^6^ Department of Radiology University of Cincinnati College of Medicine Cincinnati Ohio USA; ^7^ Department of Pediatrics University of Cincinnati College of Medicine Cincinnati Ohio USA

**Keywords:** cerebral palsy, diffusion MRI, neonatology, preterm birth, White matter

## Abstract

Infants born very preterm (VPT) are at high risk of motor impairments such as cerebral palsy (CP), and diagnosis can take 2 years. Identifying in vivo determinants of CP could facilitate presymptomatic detection and targeted intervention. Our objectives were to derive micro‐ and macrostructural measures of sensorimotor white matter tract integrity from diffusion MRI at term‐equivalent age, and determine their association with early diagnosis of CP. We enrolled 263 VPT infants (≤32 weeks gestational age) as part of a large prospective cohort study. Diffusion and structural MRI were acquired at term. Following consensus guidelines, we defined early diagnosis of CP based on abnormal structural MRI at term and abnormal neuromotor exam at 3–4 months corrected age. Using Constrained Spherical Deconvolution, we derived a white matter fiber orientation distribution (fOD) for subjects, performed probabilistic whole‐brain tractography, and segmented nine sensorimotor tracts of interest. We used the recently developed fixel‐based (FB) analysis to compute fiber density (FD), fiber‐bundle cross‐section (FC), and combined fiber density and cross‐section (FDC) for each tract. Of 223 VPT infants with high‐quality diffusion MRI data, 14 (6.3%) received an early diagnosis of CP. The cohort's mean (*SD*) gestational age was 29.4 (2.4) weeks and postmenstrual age at MRI scan was 42.8 (1.3) weeks. FD, FC, and FDC for each sensorimotor tract were significantly associated with early CP diagnosis, with and without adjustment for confounders. Measures of sensorimotor tract integrity enhance our understanding of white matter changes that antecede and potentially contribute to the development of CP in VPT infants.

## Graphical abstract

Infants born very preterm are at high risk of motor impairments such as cerebral palsy. We report biomarkers of sensorimotor white matter tract integrity, derived from diffusion MRI at term‐equivalent age, that are associated with early diagnosis of cerebral palsy.

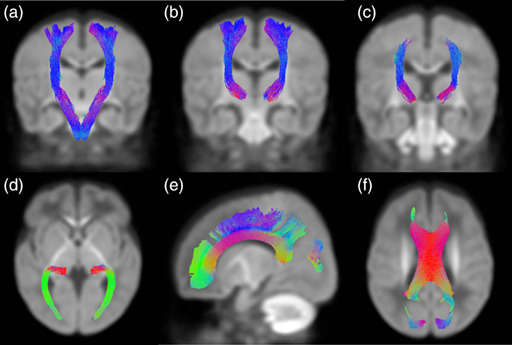

## 1 INTRODUCTION

Each year, 1 in 10 babies globally are born preterm, and those born very preterm (VPT, ≤32 weeks gestational age [GA]) face the highest risk of mortality or neurodevelopmental morbidities (Vogel et al., 2018). Of the VPT survivors, up to 50% develop mild to severe motor abnormalities such as cerebral palsy (CP), the most common physical disability in children (Spittle et al., 2011). CP prevalence is estimated at around 10% in VPT infants and increases as GA decreases (Himpens, Van den Broeck, Oostra, Calders, & Vanhaesebrouck, 2008; Vincer et al., 2006). Clinical diagnosis of CP and minor motor impairments can be delayed until 2 years of age, partially because early neuroimaging is “normal” in up to 30% of children who develop CP (Benini, Dagenais, & Shevell, 2013; de Vries, van Haastert, Benders, & Groenendaal, 2011; Hadders‐Algra, 2014; Hubermann, Boychuck, Shevell, & Majnemer, 2016). A late diagnosis leads to critical time lost for interventions at a point during brain development that is optimal for neuroplasticity. Metrics commonly used for the detection of motor impairment, including qualitative injury scores from structural magnetic resonance imaging (sMRI) and outcomes from the general movements assessment (GMA), are insufficient on their own for early, accurate diagnosis of CP (Datta et al., 2017; Hintz et al., 2015; Parikh, 2018; Van't Hooft et al., 2015). Newer prognostic imaging biomarkers available around the time of birth could promote early diagnosis and enable targeted delivery of neuroprotective interventions to preserve motor function (Parikh, 2016; Spittle, Orton, Anderson, Boyd, & Doyle, 2015).

Advanced brain MRI modalities, such as diffusion MRI (dMRI), can provide more sensitive and objective measures of motor injury. dMRI exploits the diffusion of water molecules to obtain detailed information about brain microarchitecture (Alexander, Lee, Lazar, & Field, 2007). The diffusion tensor (DT) model identifies the principal orientation of white matter fibers in three‐dimensional space, and DT metrics such as fractional anisotropy (FA) are routinely used to investigate white matter structural connectivity (Tournier, Mori, & Leemans, 2011). DT‐based tractography has illuminated the role of white matter injury in the pathophysiology of CP and motor impairments. In children with CP, injury is most often observed in the corticospinal tract (CST), posterior thalamic radiations (PTR), superior thalamic radiations (STR), and regions of the corpus callosum (CC) (Ceschin, Lee, Schmithorst, & Panigrahy, 2015; Hoon et al., 2009; Parikh, Hershey, & Altaye, 2019). However, the DT model has proven inadequate in brain regions containing crossing fibers, two or more fiber bundles with distinct orientation that contribute to a single measured signal. It is estimated that nearly 90% of white matter voxels contain crossing fibers, including the superior longitudinal fasciculus, corona radiata, PTR, and CC (Jeurissen, Leemans, Tournier, Jones, & Sijbers, 2013; Schilling et al., 2017). This poses a serious problem for DT‐based tractography methods. If spurious fiber orientations are estimated, tracking can veer off‐course, leading to false‐positive and false‐negative connections (Tournier et al., 2011). Crossing fibers also make it difficult to attribute FA, a traditional measure of tract integrity, to changes occurring at the microstructural level (Alexander, Hasan, Lazar, Tsuruda, & Parker, 2001).

Newer and more mathematically complex models have been developed to overcome limitations of the DT model and more accurately represent white matter microstructure (Jeurissen, Tournier, Dhollander, Connelly, & Sijbers, 2014; Tournier, Calamante, Gadian, & Connelly, 2004; Wang et al., 2011; Zhang, Schneider, Wheeler‐Kingshott, & Alexander, 2012). One such technique is constrained spherical deconvolution (CSD). Using high b‐shell (≥2,000) diffusion‐weighted data, CSD models the signal in each voxel as a function of all fiber population orientations present within that voxel, aka the fOD. High angular resolution diffusion imaging (HARDI) signals are thus expressed as the convolution over spherical coordinates of the response function, or the expected signal from a single population of white matter fibers, and the fOD. By performing spherical deconvolution of the diffusion signal with an estimated response function, the fOD is obtained and can be used for more accurate tractography (Tournier et al., 2004; Tournier, Calamante, & Connelly, 2007). Studies have shown the improved sensitivity and specificity of CSD‐based tractography for detecting differences in white matter diffusion characteristics, when compared to DT‐tractography (Auriat, Borich, Snow, Wadden, & Boyd, 2015; Jeurissen, Leemans, Jones, Tournier, & Sijbers, 2011; Reijmer et al., 2012). CSD is valuable in group studies; by using a single response function to compute fODs for all subjects, population‐wide differences in diffusivity can be detected (Dhollander et al., 2020; Raffelt et al., 2012).

Quantitative measures of white matter morphology derived from fODs have been proposed (Raffelt et al., 2012, 2017). These metrics are associated with single fiber populations (i.e., fibers of a single orientation) within individual voxels, also known as “fixels.” This “fixel‐based (FB) analysis,” first proposed by Raffelt et al., provides an advantage over traditional voxel‐based analysis for interpreting changes in white matter connectivity, especially in regions with crossing fibers. The mathematical framework of FB analysis allows for the calculation of fiber density (FD) and fiber‐bundle cross‐section (FC), both of which influence axonal integrity. Observed differences in FD, FC, and the combined fiber density and cross‐section (FDC), can be used to better detect aberrant axonal integrity (Raffelt et al., 2017). Histological analysis has validated the accuracy of the fOD in representing the brain's microarchitecture (Leergaard et al., 2010). Studies have also uncovered correlations between FB metrics and white matter pathology. FD and FC have been associated with damaged fiber populations in the periventricular white matter, hippocampus, cerebellum, and optic chiasm, which in some areas are consistent with histological evidence of white matter hypomyelination and disorganization (Malhotra et al., 2019; Rojas‐Vite et al., 2019).

In the preterm infant brain, white matter abnormalities are thought to result from the complex interplay between impaired axonal development and axonal degeneration (Volpe, 2009). Diffusion MRI techniques such as CSD are particularly relevant to uncovering the subtle pathology underlying the noncystic, diffuse form of periventricular leukomalacia that adversely affects the neuronal/axonal bundle but is invisible on standard anatomic MRI (Volpe, 2009). Neonatal risk factors such as bronchopulmonary dysplasia (BPD) and sepsis can also influence (e.g., delay) the microstructure of developing white matter (Rose et al., 2014). Brain injury in CP is hypothesized to follow the multifactorial etiology of preterm encephalopathy. By quantifying micro‐ and macrostructural white matter changes, FB metrics should be able to better capture aberrant white matter development following delays in development or initial direct injury to either preoligodendrocytes or immature axons with or without aberrant recovery in the pathogenesis of CP.

Our goal was to assess the micro‐ and macrostructural integrity of major sensorimotor white matter tracts in the pathophysiology of CP in a large, prospective cohort of VPT infants. To this end, we computed CSD‐derived, FB metrics from term‐equivalent age (TEA) dMRI and assessed FD, FC, and FDC as measures of tract integrity for nine sensorimotor tracts (the CC and the bilateral CST, STR–sensory, STR–motor, and PTR), which have been implicated in the development of CP (Parikh et al., 2019; Scheck, Boyd, & Rose, 2012). We hypothesized that FD, FC, and FDC of these sensorimotor tracts at TEA would be negatively associated with CP in VPT infants, diagnosed early at 3–4 months corrected age.

## 2 MATERIALS AND METHODS

### 2.1 Study design

From June 2017 to October 2019, we enrolled a prospective cohort of 263 VPT infants (≤32 weeks GA) from five level‐III neonatal intensive care units (NICUs)—Cincinnati Children's Hospital Medical Center (CCHMC), University of Cincinnati Medical Center, Good Samaritan Hospital, St. Elizabeth Healthcare, and Kettering Medical Center. Subjects were excluded if they had cyanotic heart disease or known chromosomal/congenital anomalies affecting the central nervous system. The CCHMC Institutional Review Board approved this study. A parent or guardian of each infant gave written informed consent before they were enrolled.

### 2.2 Data acquisition

MRI data collection took place at CCHMC. All subjects were imaged during natural sleep on a 3T Philips Ingenia scanner with a 32‐channel head coil between 39‐ and 44‐weeks postmenstrual age (PMA). Infants were fed 30 min prior to imaging, fitted with silicone earplugs to dampen scanner noise (Instaputty, E.A.R. Inc, Boulder, CO), and swaddled in a blanket and vacuum immobilization device (MedVac, CFI Medical Solutions, Fenton, MI). A 68 direction dMRI acquisition was performed in the axial plane with full brain coverage. Of the 68 directions, 61 had *b*‐values of 2,000 s/mm^2^ and 7 had *b*‐values of 0 s/mm^2^, with the b0s uniformly distributed throughout the acquisition (to allow for subsequent intrascan motion correction). The dMRI was acquired posterior to anterior. An additional six b0 images were acquired with anterior to posterior phase encoding in a separate acquisition. Parameters common to both acquisitions were: 88 ms echo time; 5,073 ms repetition time, flip angle 90°, field of view 160x160 mm^2^, 80 × 78 matrix, 2.0 mm slice thickness; 6:27 min scan time; multiband factor = 2; and SENSE factor = 2.

### 2.3 Global brain abnormality score

A single masked pediatric neuroradiologist performed all qualitative and quantitative MR image assessments with high reliability, as previously described (Tamm, Patel, Peugh, Kline‐Fath, & Parikh, 2020). Briefly, we used the Kidokoro scoring system (Kidokoro, Neil, & Inder, 2013) to derive a global brain abnormality score for each subject, which sums abnormalities in cortical gray matter, cerebral white matter, deep gray matter, and the cerebellum, with higher scores indicating greater abnormalities.

### 2.4 Motor testing and early diagnosis of CP

The Hammersmith Infant Neurological Examination (HINE) and Prechtl's GMA were performed at 3‐ to 4‐ months corrected age by a single masked assessor, who was unaware of clinical history or MRI results and was trained to reliability. The HINE is a standardized clinical neurological battery indicated for infants 2‐ to 24‐months of age. It generates a summed motor score (on scale 0–78) based on cranial nerve function, posture, muscle tone, and reflexes (Haataja et al., 1999; Romeo et al., 2008). The GMA is meant to identify absent or abnormal general movements of the trunk, limbs, and neck. Absence of fidgety general movements at 12‐ to 16‐ weeks corrected age strongly predicts long‐term sensorimotor impairments like CP (Einspieler & Prechtl, 2005). Using international guidelines from Novak et al. (2017), we diagnosed our high‐risk VPT infants with CP based on abnormal sMRI at TEA and the two above‐mentioned motor tests at 3‐ to 4‐months corrected age. Subjects were labeled as having a diagnosis of CP if they had any of the following combinations of abnormal prognostic tests: (a) global brain abnormality score >7 (indicating moderate or more severe brain abnormality) AND abnormal HINE exam (score <57), (b) global brain abnormality score >7 AND abnormal GMA outcome (i.e., absent fidgety movements), or (c) both abnormal HINE exam AND abnormal GMA outcome, independent of the associated brain injury score.

### 2.5 MRI preprocessing

All b2000 diffusion‐weighted data were preprocessed using MRtrix3 (www.mrtrix3.org), a CSD‐enabled software (Tournier et al., 2019) that includes calls to standard FSL (http://fsl.fmrib.ox.ac.uk/fsl/fslwiki/) preprocessing routines. Preprocessing consisted of PCA denoising and correction for Gibbs‐ringing artifacts, motion artifacts, eddy current distortions (using the reverse, anterior–posterior phase‐encoded b0 imaging data), and susceptibility‐induced off‐resonance field. Bias field correction was performed to remove low‐frequency intensity inhomogeneities, by estimating the bias present in the b0 image and using it to correct all other images for that subject. Global intensity normalization was the performed across subjects using the median b0 white matter value; we first created a temporary white matter mask for the entire population, which was then aligned to each subject's native space. A lower FA threshold of 0.15 (Vassar, Barnea‐Goraly, & Rose, 2014) was adopted to account for the higher water content of neonatal brains, and the resulting mask was checked to ensure that it covered the major white matter tracts without extending into the CSF. For more details, see MRtrix3's tutorial on calculating FB metrics using single‐tissue CSD (Tournier et al., 2019).

### 2.6 CSD and FB analysis

**FIGURE 1:**
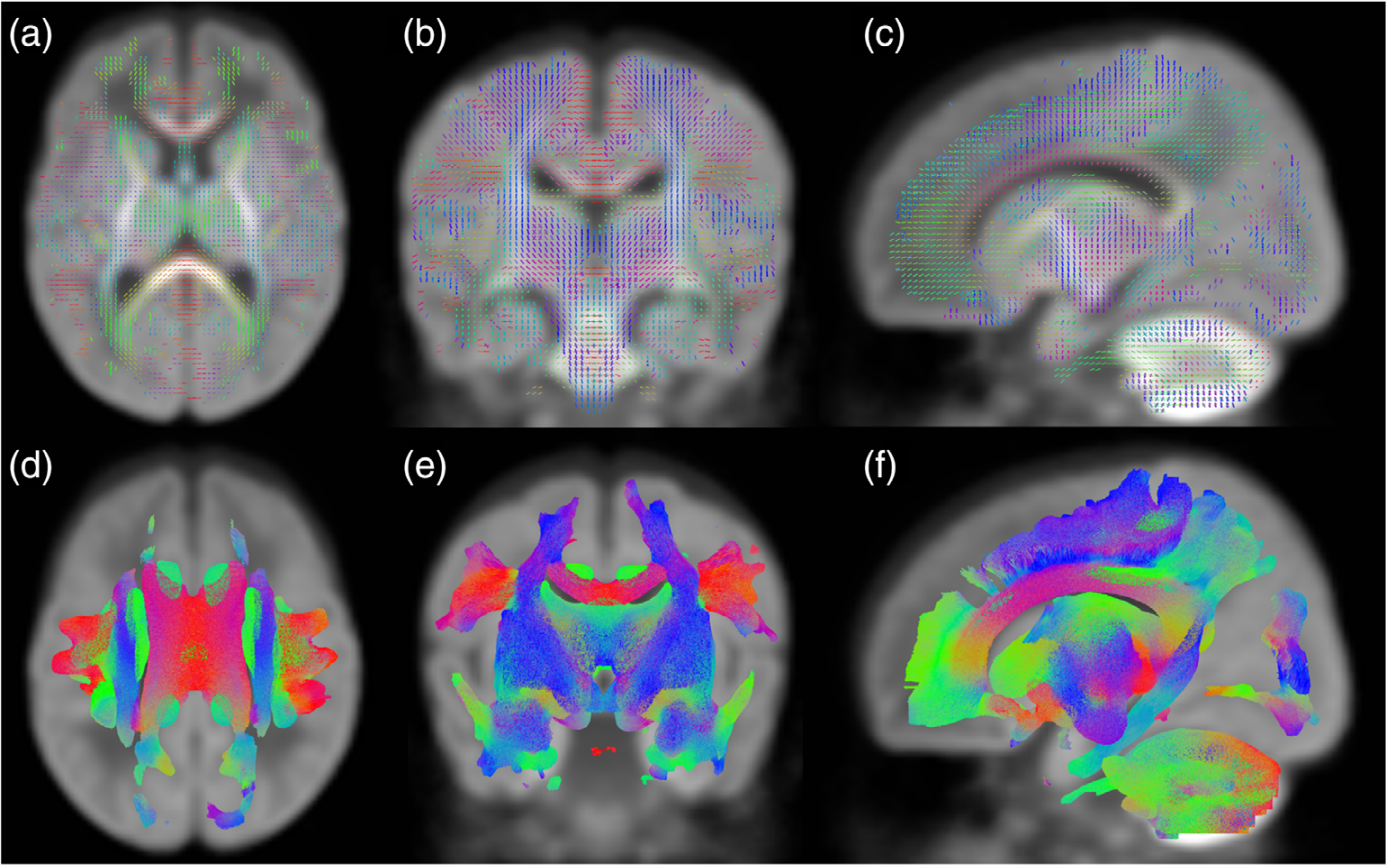
Group fixel template and whole brain tractograph used to segment sensorimotor white matter tracts. (a–c) Group average fixel map showing the fiber orientation distribution (fOD) for all voxels in axial, coronal, and sagittal views, respectively; (d–f) Corresponding whole‐brain tractograph produced from the group average fOD template. Color indicates fiber trajectory. Each tractography figure shows green (anterior to posterior), red (left to right), and blue/purple (superior to inferior) fibers

After data preprocessing, we used a custom CSD pipeline in MRtrix3 to fit a white matter fOD in each voxel for each dMRI scan. We first applied the Tournier algorithm to estimate the response function for each subject (Tournier et al., 2019). As recommended, we upsampled the preprocessed, intensity‐normalized b2000 data to an isotropic resolution of 1.3 mm^3^ (from native resolution of 2.0 mm^3^) before using the population‐average response function as the deconvolution kernel to derive a white matter fOD for each subject. A group average fOD template was generated from a subset of 40 participant (20 male and 20 female) fODs selected so that (a) the fOD did not contain any visible artifact or overt severe brain injury and (b) the PMA at MRI and GA at birth of the participant closely matched the group average values. Notably, we included a few subjects with mild ventriculomegaly (a common preterm malformation) to create the template, so that it would generalize well to the entire study cohort. The resulting fOD template was segmented to produce a group fixel template (Figure 1a–c) for use in all subsequent FB analyses, according to the methods of Raffelt et al. (2017), as implemented in MRtrix3. Each subject's fOD was then warped and registered to the template and segmented to generate fixels. We reoriented each subjects' fixels using information stored in the warp and assigned subject fixels to template fixels, to establish a 1‐to‐1 correspondence. We directly computed measures of FD, FC, and FDC (discussed in Section 2.8), and performed probabilistic whole‐brain tractography from the group fixel template.

### 2.7 Tract segmentation

We extracted nine sensorimotor tracts of interest (the CC and the bilateral CST, STR–sensory, STR–motor, and PTR) from the whole‐brain tractograph (Figure 1d–f) using a multiple region‐of‐interest (ROI) seed selection approach. In MRtrix3 ROI Editor, we created seed point, waypoint, and exclusion masks distinct to each tract, which were used to initiate tracking and to retain or exclude fibers passing through specific ROIs, respectively. We defined the initial position of the ROIs using neuroanatomical landmarks, according to our previously published methods (Kaur, Powell, He, Pierson, & Parikh, 2014; Parikh et al., 2019) and information from the group fixel template (Figure 1a–c), that is, the voxel‐wise orientation of all white matter fibers for a particular slice.

The MRtrix3 *tckedit* command allowed us to use our ROI masks in combination to generate segmented white matter tracts. The size of each ROI and the slice on which it was placed were optimized iteratively. For each iteration, the individual performing tractography varied the ROI size and position slightly and visualized the resultant tract. Through this supervised iterative process, the number of relevant fibers falsely excluded was minimized, ensuring optimization of the fibers included in each tract. Segmentation and ROI placement were performed by a single trained individual (R.C.), who was supervised and verified by the senior author who has more than 15 years of quantitative dMRI neuroimaging research experience. All tracts, except for the CC, were segmented individually for each hemisphere. Full tract extraction methods are detailed in the SI Appendix. Segmentation of the nine tracts was repeated one additional time by the first author (1 month after the initial segmentation), to evaluate intra‐rater reliability. The first segmentation attempt for each tract was used in FB analyses.

### 2.8 FD, FC, FDC

The fODs derived from CSD consist of multiple lobes representing individual fiber bundles. The amplitude of the fOD along a given fiber orientation is proportional to the radial diffusion‐weighted signal, and is therefore proportional to the intra‐axonal volume of fibers. FD is calculated by integrating the fOD of each lobe (Raffelt et al., 2012, 2017). However, a change in intra‐axonal volume may not always reflect a change in FD. Volume differences can also be accounted for by changes in morphology occurring perpendicular to the fiber orientation (i.e., a reduced fiber‐bundle cross‐section, FC). FC is the determinant of the Jacobian matrix required to spatially warp from subject to template space, with respect to fixel orientation. Last, FDC, the product of FD and FC, provides a more robust measure of axonal integrity by combining information from both metrics (Raffelt et al., 2017). For all tracts and all subjects, we generated a tract mask in template space and extracted the mean FD, FC, and FDC for fixels lying within the tract mask. These metrics served as our biomarkers of microscopic and macroscopic fiber integrity. Additionally, because white matter morphology changes rapidly during the first few months of life, we corrected our FB metrics for PMA at MRI scan according to the following formula:

(1) $$\text{Corrected}\;\mathrm{FB}\;\text{Metric}=\text{Original}\;\mathrm{FB}\;\text{Metric}+\text{slope}*\left(40-\mathrm{PMA}\right)$$

*The slope was derived from linear regression of each FB metric with PMA.

### 2.9 Statistical analyses

The intra‐rater reliability and reproducibility of tract segmentation was determined using intraclass correlation coefficients (ICC) and Dice similarity index. FB metrics were calculated for all tracts in each of two segmentation attempts (1 month apart) and then used to determine ICC. To calculate Dice similarity index, we used MRtrix3's *mrcalc* command to create an intersection mask of the two segmentations, based on the voxel‐wise overlap of the corresponding binary tract masks. Then, we quantified the number of voxels present in each tract mask, using FSL's *fslstats* command. We calculated Dice similarity index as:

(2) $$2*\text{voxels in intersection mask}/\left(\text{voxels in mask}\;1+\text{voxels in mask}\;2\right)$$

To identify confounders of sensorimotor development in our cohort, we examined group differences in variables known to be associated with CP, between study infants with and without CP diagnosis. The covariates tested are well‐established risk factors for CP, including maternal antenatal steroids and magnesium therapy, GA, sex, caffeine therapy, severe BPD (as defined by Jensen et al., 2019 based on type of respiratory support provided at 36 weeks PMA), postnatal corticosteroids for prevention/treatment of BPD, postnatal sepsis (culture‐positive), and severe retinopathy of prematurity (ROP). After assessing normality with a Shapiro–Wilk Test, we used a Mann–Whitney Rank Sum Test or a Chi Square Test, as appropriate, to identify significant group differences (*p* <.05 indicated statistical significance). These tests were also used to assess statistical differences in baseline variables between the excluded subjects and the final cohort used in the analysis. The same baseline variables were compared across the representative subset of 40 subjects used to create the fOD template and the total population.

We used logistic regression analysis to determine the relationship of PMA‐corrected FB metrics for each tract of interest and early CP diagnosis, with and without controlling for the significant confounders. When Benjamini–Hochberg false discovery rate (FDR) correction (Benjamini & Hochberg, 1995) was applied to regression p values (accounting for 27 comparisons made), all relationships remained significant (Table 2). This was true when FDR correction was applied before or after covariate adjustment. In a separate analysis for infants without CP diagnosis (aka the low‐risk group), we used linear regression to determine the relationship of PMA‐corrected FB metrics for each tract of interest and HINE score. Statistical analyses were conducted in Stata version 13.1, unless otherwise specified.

## 3 RESULTS

### 3.1 Demographics

Of the initial cohort of 263 infants with b2000 dMRI acquired data, six subjects were excluded at the global intensity normalization step, due to bright or dark artifacts that interfered with normalization across all subjects. Twenty‐three additional subjects were excluded because they were missing small brain regions at the periphery of their diffusion‐weighted scans (necessary because a 1‐to‐1 correspondence is required between subject and template fixels, and FB analysis in MRtrix3 can only be performed for brain regions shared by all subjects). Two subjects were excluded due to suboptimal alignment with the group fOD template, and two more were excluded with extreme ventriculomegaly causing poor registration of the fOD to the template. Of the remaining 230 infants with completed CSD analysis, 223 (97%) had the follow‐up data at 3–4 months corrected age necessary to generate CP diagnosis, and they constituted the final cohort. Their mean (*SD*) GA and PMA at MRI scan were 29.4 (2.4) and 42.8 (1.3) weeks, respectively. Mean (*SD*) birth weight was 1,279 (433) grams. Apart from sex, there were no statistically significant differences in baseline variables between the 40 infants excluded (mean [*SD*] GA of 29.5 [2.7] weeks, PMA at MRI of 42.4 [1.3] weeks, birth weight of 1384 [459] grams, and 65% male) and the final cohort of 223 infants. The subset of 40 subjects used to create the fOD template (mean [SD] GA of 29.3 [2.7] weeks, PMA at MRI scan of 42.9 [1.2] weeks, birth weight of 1256 [391] grams, and 50% male) was representative of the larger cohort without any statistically significant group differences.

Our final cohort had a median HINE score of 60 (IQR: 6; range: 30.5–70); 54 (24.2%) infants had an abnormal HINE score of <57. Nine infants (4.0%) had an abnormal GMA exam, and 22 (9.9%) infants had moderate to severe brain abnormality on sMRI. Our cohort included seven infants with both abnormal MRI and HINE, four with both abnormal HINE and GMA, and three with both abnormal MRI and GMA. Overall, 14 (6.3%) VPT infants received an early diagnosis of CP based on our a priori definition (Novak et al., 2017). Demographic and clinical variables for infants with CP diagnosis and those without diagnosis (low‐risk for CP) are shown in Table 1.

**TABLE 1:** Baseline demographics and clinical characteristics of very preterm infants with and without early diagnosis of cerebral palsy (CP)

Clinical variables^a^	Low risk CP (*N* = 209)	Early CP (*N* = 14)	*p* value
Maternal antenatal steroids, *N* (%)	195 (93.3%)	11 (78.6%)	.079
Maternal magnesium therapy, *N* (%)	175 (83.7%)	10 (71.4%)	.380
Gestational age at birth, weeks	29.4 (2.4)	28.2 (2.8)	.100
Birth weight, grams	1,292.1 (429.9)	1,091.0 (454.0)	.143
Male, *N* (%)	98 (46.9%)	6 (42.9%)	.770
Head circumference at birth (cm)	26.7 (2.9)	25.1 (3.8)	.189
Postnatal corticosteroids, *N* (%)	17 (8.1%)	4 (28.6%)	*.031*
Sepsis (culture positive), *N* (%)	16 (7.7%)	5 (35.7%)	*.001*
Caffeine therapy, *N* (%)	154 (73.7%)	8 (57.1%)	.179
Severe retinopathy of prematurity, *N* (%)	8 (3.8%)	1 (7.1%)	.448
Severe bronchopulmonary dysplasia, *N* (%)	28 (13.4%)	6 (42.9%)	*.003*
Postmenstrual age at MRI scan	42.7 (1.3)	43.3 (1.0)	.150

*Note*: Values in italics (p < 0.05) indicates statistical significance.

a All values are mean (*SD*) unless otherwise indicated.

### 3.2 Covariate selection

Between‐group analyses for infants with CP diagnosis (*n* = 14) and without CP diagnosis revealed significant differences in severe BPD, postnatal corticosteroids for BPD, and postnatal sepsis (Table 1). Several variables, including severe BPD and postnatal sepsis, are well‐known neonatal risk factors that have been shown to influence white matter microstructure (Rose et al., 2014). These potential confounders, along with GA, were included as covariates in all logistic regression analyses. As expected, severe BPD, postnatal corticosteroid use, and postnatal sepsis were associated with increased CP risk. There were no statistically significant differences in sex, antenatal steroids, maternal magnesium therapy, caffeine therapy, and severe ROP between groups.

### 3.3 FD, FC, FDC associations with motor outcomes

**FIGURE 2:**
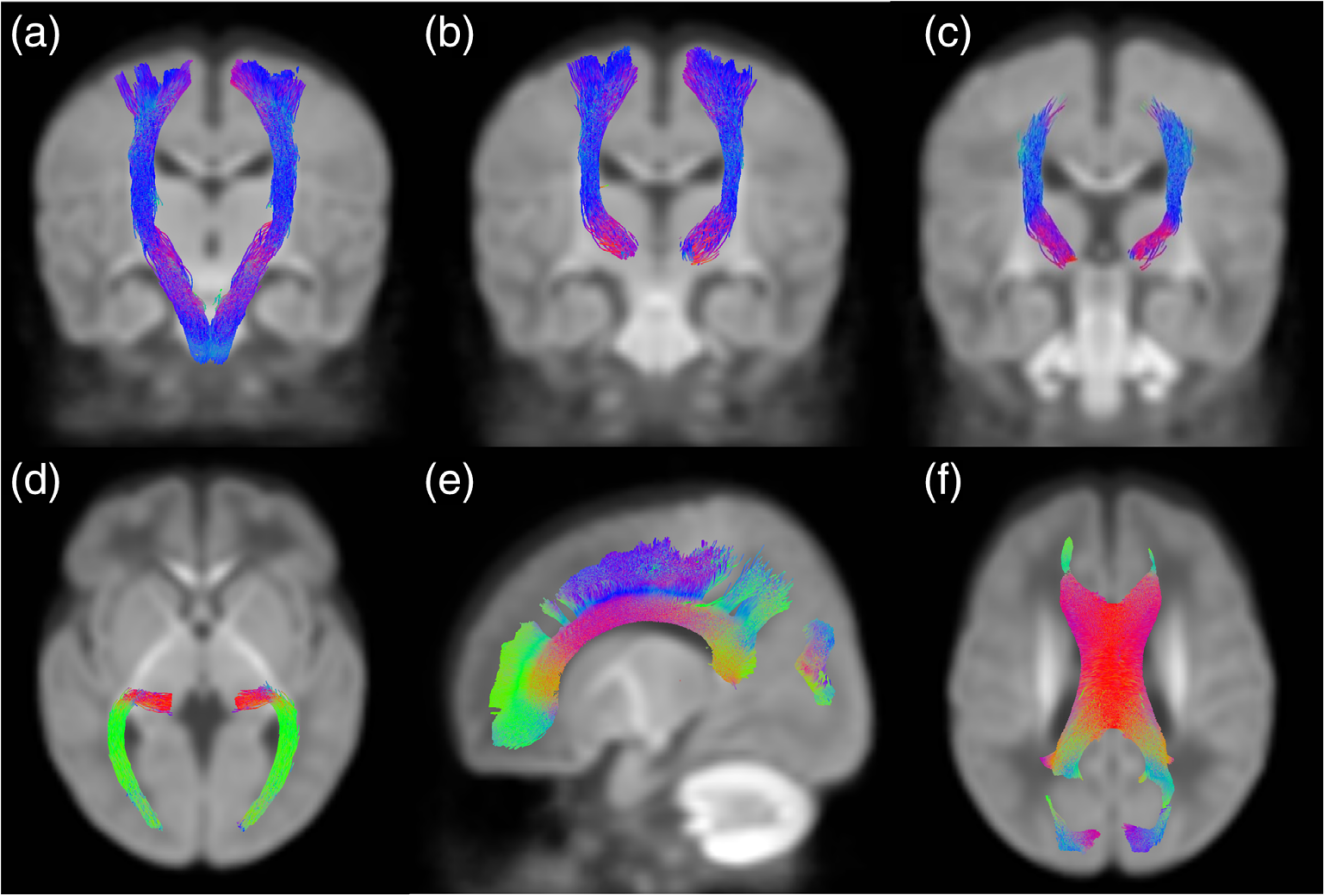
Bilateral and unilateral segmentations of sensorimotor tracts. (a) Corticospinal tract in coronal view; (b) superior thalamic radiations (motor) in coronal view; (c) superior thalamic radiations (sensory) in coronal view; (d) posterior thalamic radiations in axial view; (e) corpus callosum in sagittal view; (f) corpus callosum in axial view. All fibers of the STRS and CC are not visible in a single view, as the tracts continue from the thalamus to the postcentral gyrus and from the splenium to the posterior brain, respectively. Color indicates fiber trajectory. Each tractography figure shows green (anterior to posterior), red (left to right), and blue/purple (superior to inferior) fibers

Figure 2 shows the final unilateral or bilateral segmentations for the CST, STR (motor and sensory), PTR, and CC. FB metrics were calculated for each subject and tract, and the mean (*SD*) values are shown in Table S1; we found widespread significant differences in mean FB metrics between groups. Tables 2, S2, and S3 display the results of logistic regression analyses correlating FDC, FD, and FC, respectively with early CP diagnosis, both with and without covariate correction.

**TABLE 2:** Logistic regression analysis between sensorimotor tract fiber density and cross‐section (FDC) and cerebral palsy, with and without adjustment for clinical confounders

	FB metrics corrected for PMA at MRI only				FB metrics corrected for PMA at MRI and confounders^a^			
	Coef. (95% CI)	Odds ratio	*p* value	Pseudo R2	Coef. (95% CI)	Odds ratio	*p* value	Pseudo R2
*RCST*	−17.967 (−26.058, −9.8759)	1.57e−08	*<.001*	0.228	−16.223 (−24.731, −7.716)	9.00e−08	*<.001*	0.264
Severe BPD					0.531 (−1.380, 2.442)	1.700	.586	
Steroids BPD					0.393 (−1.902, 2.688)	1.481	.737	
Sepsis					0.746 (−0.853, 2.346)	2.110	.360	
GA					−0.131 (−0.301, 0.275)	0.987	.929	
*LCST*	−19.392 (−27.566, −11.218)	3.79e−09	*<.001*	0.269	−17.838 (−26.339, −9.336)	1.79e−08	*<.001*	0.303
Severe BPD					0.526 (−1.418, 2.469)	1.692	.596	
Steroids BPD					0.175 (−2.189, 2.540)	1.192	.884	
Sepsis					0.885 (−0.733, 2.503)	2.424	.284	
GA					−0.025 (−0.320, 0.270)	0.975	.869	
*RSTRM*	−16.622 (−24.881, −8.362)	6.04e−08	*<.001*	0.183	−14.710 (−23.360, −6.061)	4.09e−07	*.001*	0.230
Severe BPD					0.713 (−1.100, 2.526)	2.041	.441	
Steroids BPD					0.258 (−1.976, 2.491)	1.294	.821	
Sepsis					0.787 (−0.759, 2.332)	2.196	.318	
GA					−0.024 (−0.306, 0.258)	0.976	.868	
*LSTRM*	−18.633 (−27.076, −10.190)	8.08e−09	*<.001*	0.221	−16.874 (−25.684, −8.065)	4.69e−08	*<.001*	0.265
Severe BPD					0.665 (−1.159, 2.489)	1.944	.475	
Steroids BPD					0.076 (−2.200, 2.353)	1.079	.948	
Sepsis					0.916 (−0.627, 2.459)	2.500	.245	
GA					−0.038 (−0.327, 0.251)	0.962	.795	
*RSTRS*	−18.720 (−27.550, −9.890)	7.42e−09	*<.001*	0.202	−16.884 (−26.188, −7.581)	4.65e−08	*<.001*	0.245
Severe BPD					0.616 (−1.211, 2.443)	1.851	.509	
Steroids BPD					0.329 (−1.915, 2.572)	1.389	.774	
Sepsis					0.831 (−0.718, 2.379)	2.294	.293	
GA					−0.014 (−0.302, 0.274)	0.986	.925	
*LSTRS*	−21.381 (−30.508, −12.255)	5.18e−10	*<.001*	0.258	−19.749 (−29.193, −10.306)	2.65e−09	*<.001*	0.297
Severe BPD					0.552 (−1.341, 2.445)	1.737	.568	
Steroids BPD					0.064 (−2.278, 2.406)	1.066	.957	
Sepsis					0.967 (−0.613, 2.548)	2.631	.230	
GA					−0.026 (−0.322, 0.270)	0.974	.863	
*RPTR*	−23.617 (−34.083, −13.151)	5.54e−11	*<.001*	0.225	−20.925 (−31.953, −9.896)	8.17e−10	*<.001*	0.247
Severe BPD					0.542 (−1.234, 2.317)	1.719	.550	
Steroids BPD					0.118 (−2.052, 2.288)	1.125	.915	
Sepsis					0.481 (−1.075, 2.037)	1.618	.545	
GA					−0.043 (−0.334, 0.247)	0.958	.771	
*LPTR*	−23.557 (−34.224, −12.889)	5.88e−11	*<.001*	0.224	−20.476 (−31.672, −9.280)	1.28e−09	*<.001*	0.243
Severe BPD					0.429 (−1.354, 2.212)	1.536	.637	
Steroids BPD					−0.108 (−2.275, 2.060)	0.898	.923	
Sepsis					0.757 (−0.760, 2.273)	2.131	.328	
GA					−0.022 (−0.307, 0.262)	0.978	.879	
*CC*	−21.369 (−31.507, −11.230)	5.24e−10	*<.001*	0.191	−19.097 (−30.109, −8.086)	5.08e−09	*.001*	0.219
Severe BPD					0.834 (−0.864, 2.531)	2.302	.336	
Steroids BPD					−0.163 (−2.271, 1.945)	0.849	.879	
Sepsis					0.658 (−0.866, 2.182)	1.930	.398	
GA					0.022 (−0.255, 0.299)	1.022	.875	

*Note*: Values in italics (*p* < .05) indicates statistical significance.

Abbreviations: CC, corpus callosum; corticospinal tract (right, RCST; left, LCST); FB, fixel‐based; PMA, postmenstrual age; posterior thalamic radiations (right, RPTR, left, LPTR); superior thalamic radiations motor (right, RSTRM, left, LSTRM); superior thalamic radiations sensory (right, RSTRS, left, LSTRS).

a Confounders included gestational age, severe bronchopulmonary dysplasia, postnatal corticosteroids, and postnatal sepsis.

In univariate analysis, FD, FC, and FDC for all nine sensorimotor tracts were negatively associated with CP. This remained true when all significant confounders were included in the model, demonstrating the independent significance of these FB metrics on development of CP. These results suggest that axonal integrity and myelination in multiple sensorimotor tracts are important in the pathophysiology of CP.

For infants in the low‐risk CP group, we found significant, positive associations between HINE score and FDC of the right CST, right STRM, bilateral STRS, bilateral PTR, and CC, prior to adjustment for confounders. After including covariates in the model, only FDC of the bilateral PTR remained significant (Table 3). Regression analysis for FDC versus each individual test—abnormal sMRI, HINE, and GMA—are shown in Tables S4–S6.

**TABLE 3:** Linear regression analysis between sensorimotor tract fiber density and cross‐section (FDC) and HINE for infants with low‐risk CP, with and without adjustment for clinical confounders

	FB metrics corrected for PMA at MRI only			FB metrics corrected for PMA at MRI and confounders^a^		
	Coef. (95% CI)	*p* value	Adj. *R* ^2^	Coef. (95% CI)	*p* value	Adj. *R* ^2^
*RCST*	10.262 (0.711, 19.812)	*.035*	0.017	7.155 (−2.293, 16.603)	.137	0.066
Severe BPD				−0.265 (−2.633, 2.103)	.826	
Steroids BPD				−1.398 (−4.481, 1.686)	.372	
Sepsis				−2.860 (−5.483, −0.237)	*.033*	
GA				0.210 (−0.136, 0.555)	.233	
*LCST*	8.821 (−1.080, 18.722)	.080	0.010	5.739 (−4.028, 15.506)	.248	0.062
Severe BPD				−0.264 (−2.638, 2.109)	.826	
Steroids BPD				−1.388 (−4.480, 1.704)	.377	
Sepsis				−2.916 (−5.543, −0.289)	*.030*	
GA				0.216 (−0.129, 0.562)	.218	
*RSTRM*	10.360 (0.989, 19.731)	*.030*	0.018	7.774 (−1.452, 17.001)	.098	0.068
Severe BPD				−0.257 (−2.622, 2.108)	.830	
Steroids BPD				−1.399 (−4.478, 1.680)	.371	
Sepsis				−2.822 (−5.444, −0.200)	*.035*	
GA				0.218 (−0.126, 0.562)	.214	
*LSTRM*	9.313 (−0.797, 19.424)	.071	0.011	6.735 (−3.188, 16.658)	.182	0.064
Severe BPD				−0.253 (−2.623, 2.118)	.834	
Steroids BPD				−1.386 (−4.473, 1.702)	.377	
Sepsis				−2.905 (−5.528, −0.283)	*.030*	
GA				0.221 (−0.124, 0.566)	.207	
*RSTRS*	13.287 (2.727, 23.848)	*.014*	0.024	9.966 (−0.482, 20.414)	.061	0.072
Severe BPD				−0.202 (−2.563, 2.158)	.866	
Steroids BPD				−1.430 (−4.502, 1.643)	.360	
Sepsis				−2.844 (−5.455, −0.232)	*.033*	
GA				0.204 (−0.140, 0.548)	.244	
*LSTRS*	12.343 (1.121, 23.565)	*.031*	0.018	8.679 (−2.422, 19.780)	.125	0.067
Severe BPD				−0.232 (−2.599, 2.135)	.847	
Steroids BPD				−1.382 (−4.465, 1.700)	.378	
Sepsis				−2.874 (−5.494, −0.254)	*.032*	
GA				0.211 (−0.134, 0.556)	.229	
*RPTR*	27.040 (13.178, 40.902)	*<.001*	0.062	19.862 (5.324, 34.400)	*.008*	0.088
Severe BPD				−0.245 (−2.585, 2.094)	.836	
Steroids BPD				−0.750 (−3.841, 2.340)	.633	
Sepsis				−2.330 (−4.963, 0.302)	.082	
GA				0.219 (−0.121, 0.559)	.206	
*LPTR*	23.697 (10.126, 37.268)	*.001*	0.050	16.449 (2.294, 30.603)	*.023*	0.079
Severe BPD				−0.125 (−2.477, 2.228)	.917	
Steroids BPD				−0.945 (−4.038, 2.147)	.547	
Sepsis				−2.543 (−5.171, 0.086)	.058	
GA				0.213 (−0.128, 0.555)	.220	
*CC*	19.897 (7.518, 32.277)	*.002*	0.042	12.339 (−0.729, 24.407)	.064	0.071
Severe BPD				−0.250 (−2.611, 2.111)	.835	
Steroids BPD				−1.018 (−4.128, 2.092)	.519	
Sepsis				−2.558 (−5.213, 0.096)	.059	
GA				0.203 (−0.141, 0.547)	.246	

*Note*: Values in italics (*p* < .05) indicates statistical significance.

Abbreviations: CC, corpus callosum; corticospinal tract (right, RCST, left, LCST); FB, fixel‐based; PMA, postmenstrual age; posterior thalamic radiations (right, RPTR, left, LPTR); superior thalamic radiations motor (right, RSTRM, left, LSTRM); superior thalamic radiations sensory (right, RSTRS, left, LSTRS).

a Confounders included gestational age, severe bronchopulmonary dysplasia, postnatal corticosteroids, and postnatal sepsis.

### 3.4 Reliability and reproducibility

Intra‐rater reliability, measured via ICC and Dice similarity index, is displayed in Table S7. The ICC for the FDC metric ranged from 0.9974 to 0.9999 across the nine tracts. The Dice similarity index, calculated via voxel‐wise tract overlap, ranged from 0.9221 to 0.9908. High ICC and dice similarity values indicate that our tractography protocol is reliable and reproducible.

## 4 DISCUSSION

We have demonstrated that CSD‐derived, FB measures of white matter integrity for key sensorimotor tracts are independently associated with early diagnosis of CP in VPT infants at TEA. FB metrics—FD, FC, and FDC—for each of the nine tracts examined were negatively and strongly associated with CP in unadjusted and adjusted analyses. This is a novel finding, as no prior study has reported such robust, consistent associations between measures of micro and macroscopic fiber integrity at TEA and early motor outcomes. Our findings demonstrate the role of these tracts in the pathophysiology and functional development of CP.

Using the FB framework of Raffelt et al. (2017), we obtained a more comprehensive understanding of how pathophysiological changes in fiber morphometry influence aberrant motor development in VPT infants. Previous studies have shown that pathology and abnormal brain development alter diffusivity and induce changes in fiber response function (i.e., the diffusion‐weighted signal of a single fiber population; Beaulieu, 2002; Tournier et al., 2004). Because we used a population‐averaged response function for CSD, differences between the average response function and individual subject response functions are reflected as differences in fOD amplitude, and therefore, as differences in FD (Raffelt et al., 2012; Tournier et al., 2004). In our study, we examined relative FD values as an indicator of axonal integrity. Higher FD values for our infants likely reflect a larger intra‐axonal volume, either via a greater number of axons or via increased axonal diameter within a voxel (Raffelt et al., 2017). Histological analyses have shown that FD is correlated with axonal density and successfully identifies damaged fiber populations, particularly in crossing fiber regions like the optic chiasm (Rojas‐Vite et al., 2019).

In addition to within‐voxel tract density, FC allows us to query relative differences in macroscopic tract morphology. A change in the number of voxels a fiber bundle occupies impacts its volume and information transfer capacity, making FC a separate but complementary measure of tract integrity. Lower FC values in infants may represent thinner fiber bundles at TEA, possibly due to decreased myelination or white matter atrophy after the deposition of extracellular matrix and inflammatory cells into the extra‐axonal space (Malhotra et al., 2019; Raffelt et al., 2017). Raffelt et al. also proposed that FDC, accounting for the complex interdependency of the changes in FD and FC, provides a more sensitive measure of intra‐axonal volume and thus, a tract's ability to relay information. In our cohort, decreases in all three metrics were independently associated with early CP diagnosis, allowing us to assert that the pathophysiology of CP in VPT infants involves widespread decreased axonal integrity of sensorimotor tracts via diminished within‐voxel FD and tract FC.

We identified significant negative associations between FB metrics and CP diagnosis in nine sensorimotor tracts, including the CC, and the bilateral CST, STR (motor and sensory), and PTR. Prior pilot DT‐based studies have implicated injury or immaturity to various tracts in white matter abnormalities found in preterm infants and children with CP (Pannek, Scheck, Colditz, Boyd, & Rose, 2014; Parikh et al., 2019; Scheck et al., 2012). Our results support these findings and further demonstrate the importance of all nine tracts in early motor development. Several studies have shown differences in DT‐derived tract metrics between preterm and term infants, but few have reported on the relationship between DT metrics and early motor outcomes. The CST is a major pathway carrying voluntary motor information. Parikh et al. (2019) found significant differences in FA and radial diffusivity (RD) of the CST at TEA between 36 extremely‐low‐birth‐weight preterm infants without and 5 infants with CP at 18–22 months corrected age. Other studies (De Bruine et al., 2013; Rose et al., 2015; van Kooij et al., 2011) report associations between the FA or mean diffusivity (MD) of the PLIC (a CST region of interest) and Bayley‐III motor outcomes. Another tract, the STR, relays motor information from the thalamus to the cerebral cortex. Berman et al. (2005) performed fiber tracking of 27 preterm infants and found that FA of the somatosensory tracts (part of the STR) increased with increasing age. Parikh et al. (2019) found similar, large differences in DT metrics in the STRM and STRS between infants with and without CP. The CC is likewise critical to controlling motor function, being responsible for transferring information across the hemispheres. Our findings corroborate prior studies (Barnett et al., 2017; Parikh et al., 2019; Thompson et al., 2011) showing reduced FA or increased RD and MD of the CC in at‐risk infants, corresponding to worse motor outcomes. The PTR connects the thalamus to the parietal and occipital lobes and has been implicated in motor function related to proprioception and touch threshold (Hoon et al., 2009). In our analysis, FB metrics of the PTR were significantly associated with CP diagnosis. Studies have reported mixed results regarding the PTR in motor development, with a prior pilot study (Parikh et al., 2019) finding no association between DT metrics of the PTR and CP, and others finding a negative association between FA in older children and CP (Yoshida et al., 2010, 2011).

Collectively, our findings are consistent with these smaller studies. However, individually, many of them report associations in only a few ROIs or full sensorimotor tracts, while we report widespread associations across all nine tracts examined. This discrepancy likely results from the higher statistical power of our large study and the higher sensitivity of CSD with HARDI‐acquired data as a method for investigating white matter tract integrity. To our knowledge, only one other study (Pannek et al., 2020) has investigated FB metrics of sensorimotor tracts in relation to motor outcomes in VPT infants. Pannek et al. (2020) recently reported that standardized motor scores at 1‐year corrected age were positively associated with FD, FC, and FDC for the right CST; FD and FDC for the right PTR; FDC in the left CST; and were negatively associated with FC in the genu of the CC. At 1‐year, they found no association with FC for the right PTR; FD and FC for the left CST; FD and FDC for the genu of the CC; and any FB metric for the left PTR or splenium of the CC. They did not report CP analyses/outcomes. Overall, their findings suggest that only a few integrity metrics are associated with motor outcomes for some tracts. However, in our analyses, we demonstrated consistent associations between early CP diagnosis and FD, FC, and FDC for each of the nine examined tracts. Pannek et al. also investigated DT‐based measures and motor scores, but did not find significant associations between FA, MD, RD, or axial diffusivity (AD) of any full tract and motor scores at 1‐year corrected age. Numerous studies, including those mentioned previously, have established clear relationships between DT‐based measures of sensorimotor tracts and motor outcomes. There are several possible explanations for why our results differ from Pannek et al. and ultimately reveal a broader range of associations across the FB metrics and tracts. First, Pannek et al. studied a smaller cohort, consisting of 78 VPT infants, which significantly limits statistical power compared to our larger cohort. Pannek et al. also did not report the methodology used to segment their sensorimotor tracts from CSD. In contrast, we report an extensive, iterative process used to optimize the segmentation accuracy of tracts. The ICC and Dice similarity index across our two segmentations support the reliability of our results. While Pannek et al. used motor scores on the Bayley Scales of Infant and Toddler Development as their outcome, which represents a range of mild to severe motor development, we correlated FB metrics with a more severe motor outcome, early CP diagnosis, which is based on a combination of abnormalities on neuroimaging and motor outcomes (Novak et al., 2017). Furthermore, nearly 40% of preterm infants who do not develop CP can have mild to moderate motor impairments (Williams, Lee, & Anderson, 2010). In a separate analysis, we found significant positive associations between FB metrics of sensorimotor tracts and HINE score in our cohort's low‐risk group (Table 3). This finding is unique and suggests that micro and macrostructural metrics of the neonatal brain at term may have the potential to detect milder motor impairments than CP, which may nevertheless benefit from early intervention.

Our study had a number of strengths, including our large, multi‐center, regional cohort of 223 VPT infants. We used comprehensive methods that queried the major sensorimotor tracts that are negatively impacted in older children with CP. Using CSD and FB analysis to assess the integrity of white matter tracts was a major strength of the study. An estimated 90% of voxels contain crossing fibers, which make it difficult to accurately interpret DT‐based analyses, as changes at the microstructural level may be undetected or misinterpreted due to a lack of fiber specificity. FB metrics have higher sensitivity than voxel‐wise DT metrics in detecting group‐wide differences in white matter (Dhollander et al., 2020). We also used meticulous tract‐extraction methods based on previously‐published methods (Kaur et al., 2014; Parikh et al., 2019; Teli et al., 2018) and our own systematic, iterative process. The high correlation between our FB metrics across all sensorimotor tracts and the high intra‐rater reliability of the segmentation attest to the quality of our methods. Finally, we adjusted our logistic regression analyses for several confounders that are known to influence motor development, thus demonstrating the independent significance of these FB metrics in the development of CP.

There are several limitations to our study. To facilitate early detection of CP, we used the Novak international early CP diagnosis guideline that combines sMRI at TEA with 3 to 4‐month corrected age HINE or GMA scores (Novak et al., 2017). These tests represent the earliest and most predictive tests of motor outcomes available and are used clinically by many centers. However, this combination of tests has yet to be independently validated with later diagnosis of CP (Parikh, 2018). Moreover, our findings suggest that our early CP definition and/or FB metrics may lack the ability to differentiate the type/severity of CP. However, this question can only be answered once we correlate FB metrics (in comparison with DT‐based metrics) with more confirmatory diagnosis at 2 years corrected age, as we are currently doing. Prior studies using DT‐based metrics have shown an association between FA from the CST and CP severity (Cho et al., 2013; Rose et al., 2007). Nevertheless, infants with a combination of abnormal tests are at higher risk for developing CP. An advantage of using this guideline for early diagnosis is that it is less confounded by early intervention therapies and treatments/exposures following NICU discharge and before age 2. We lacked an analysis of targeted ROIs within tracts, which could reveal more detailed information about the underlying associations. Finally, while the present analyses focused on investigating the association between FB metrics and CP, the results indicate, to an extent, the potential value of these metrics for predicting motor outcomes. Brain morphometric biomarkers that we and others have reported, such as brain volumes and cortical surface measures (Dubois et al., 2008; Kline, Illapani, He, Altaye, et al., 2020; Kline, Illapani, He, & Parikh, 2020; Parikh et al., 2020), may be used in conjunction with FB metrics to enhance prediction of motor outcomes.

## 5 CONCLUSION

In summary, we have shown that CSD derived, FB measures of axonal integrity from key sensorimotor tracts at TEA are independently associated with the early diagnosis of CP in VPT infants. This is an important finding, as it demonstrates the role of multiple sensorimotor tracts in the pathophysiology of CP and related motor impairments. VPT infants remain at high risk of motor impairments, and there is an urgent need to establish validated biomarkers that can be used for early detection and intervention.

## CONFLICT OF INTEREST

The authors declare no potential conflict of interest.

## Supporting information

**AppendixS1:** Supporting Information

## Data Availability

Datasets generated from this study and code used in the analysis are available from the corresponding author upon request.

## References

[hbm25579-bib-0001] Alexander, A. L., Hasan, K. M., Lazar, M., Tsuruda, J. S., & Parker, D. L. (2001). Analysis of partial volume effects in diffusion‐tensor MRI. Magnetic Resonance in Medicine, 45(5), 770–780. 10.1002/mrm.1105 11323803

[hbm25579-bib-0002] Alexander, A. L., Lee, J. E., Lazar, M., & Field, A. S. (2007). Diffusion tensor imaging of the brain. Neurotherapeutics: The Journal of the American Society for Experimental NeuroTherapeutics, 4(3), 316–329. 10.1016/j.nurt.2007.05.011 17599699 PMC2041910

[hbm25579-bib-0003] Auriat, A. M., Borich, M. R., Snow, N. J., Wadden, K. P., & Boyd, L. A. (2015). Comparing a diffusion tensor and non‐tensor approach to white matter fiber tractography in chronic stroke. NeuroImage: Clinical, 7, 771–781. 10.1016/j.nicl.2015.03.007 25844329 PMC4375634

[hbm25579-bib-0004] Barnett, M. L., Tusor, N., Ball, G., Chew, A., Falconer, S., Aljabar, P., … Counsell, S. J. (2017). Exploring the multiple‐hit hypothesis of preterm white matter damage using diffusion MRI. NeuroImage: Clinical, 17, 596–606. 10.1016/j.nicl.2017.11.017 29234596 PMC5716951

[hbm25579-bib-0005] Beaulieu, C. (2002). The basis of anisotropic water diffusion in the nervous system ‐ A technical review. NMR in Biomedicine, 15(7–8), 435–455. 10.1002/nbm.782 12489094

[hbm25579-bib-0006] Benini, R., Dagenais, L., & Shevell, M. I. (2013). Normal imaging in patients with cerebral palsy: What does it tell us? The Journal of Pediatrics, 162(2), 369–374. 10.1016/j.jpeds.2012.07.044 22944004

[hbm25579-bib-0007] Benjamini, Y., & Hochberg, Y. (1995). Controlling the false discovery rate: A practical and powerful approach to multiple testing. Journal of the Royal Statistical Society, 57, 289–300. 10.2307/2346101

[hbm25579-bib-0008] Berman, J. I., Mukherjee, P., Partridge, S. C., Miller, S. P., Ferriero, D. M., Barkovich, A. J., … Henry, R. G. (2005). Quantitative diffusion tensor MRI fiber tractography of sensorimotor white matter development in premature infants. NeuroImage, 27(4), 862–871. 10.1016/j.neuroimage.2005.05.018 15978841

[hbm25579-bib-0009] Ceschin, R., Lee, V. K., Schmithorst, V., & Panigrahy, A. (2015). Regional vulnerability of longitudinal cortical association connectivity: Associated with structural network topology alterations in preterm children with cerebral palsy. NeuroImage: Clinical, 9, 322–337. 10.1016/j.nicl.2015.08.021 26509119 PMC4588423

[hbm25579-bib-0010] Cho, H. K., Jang, S. H., Lee, E., Kim, S. Y., Kim, S., Kwon, Y. H., & Son, S. M. (2013). Diffusion tensor imaging–demonstrated differences between hemiplegic and Diplegic cerebral palsy with symmetric periventricular Leukomalacia. AJNR. American Journal of Neuroradiology, 34(3), 650–654. 10.3174/ajnr.A3272 22976239 PMC7964924

[hbm25579-bib-0011] Datta, A. N., Furrer, M. A., Bernhardt, I., Hüppi, P. S., Borradori‐Tolsa, C., Bucher, H. U., … Group, G.M . (2017). Fidgety movements in infants born very preterm: Predictive value for cerebral palsy in a clinical multicentre setting. Developmental Medicine and Child Neurology, 59(6), 618–624. 10.1111/dmcn.13386 28102574

[hbm25579-bib-0012] De Bruine, F. T., Van Wezel‐Meijler, G., Leijser, L. M., Steggerda, S. J., Van Den Berg‐Huysmans, A. A., Rijken, M., … Van Der Grond, J. (2013). Tractography of white‐matter tracts in very preterm infants: A 2‐year follow‐up study. Developmental Medicine and Child Neurology, 55(5), 427–433. 10.1111/dmcn.12099 23441853

[hbm25579-bib-0013] de Vries, L. S., van Haastert, I. C., Benders, M. J., & Groenendaal, F. (2011). Myth: Cerebral palsy cannot be predicted by neonatal brain imaging. Seminars in Fetal and Neonatal Medicine, 16(5), 279–287. 10.1016/j.siny.2011.04.004 21636334

[hbm25579-bib-0014] Dhollander, T., Clemente, A., Singh, M., Boonstra, F., Civier, O., Duque, J., … Caeyenberghs, K. (2020). Fixel‐based Analysis of Diffusion MRI: Methods, Applications, Challenges and Opportunities. 10.31219/osf.io/zu8fv 34298083

[hbm25579-bib-0015] Dubois, J., Benders, M., Borradori‐Tolsa, C., Cachia, A., Lazeyras, F., Ha‐Vinh Leuchter, R., … Hüppi, P. S. (2008). Primary cortical folding in the human newborn: An early marker of later functional development. Brain: A Journal of Neurology, 131(8), 2028–2041. 10.1093/brain/awn137 18587151 PMC2724902

[hbm25579-bib-0016] Einspieler, C., & Prechtl, H. F. (2005). Prechtl's assessment of general movements: A diagnostic tool for the functional assessment of the young nervous system. Mental Retardation and Developmental Disabilities Research Reviews, 11(1), 61–67. 10.1002/mrdd.20051 15856440

[hbm25579-bib-0017] Haataja, L., Mercuri, E., Regev, R., Cowan, F., Rutherford, M., Dubowitz, V., & Dubowitz, L. (1999). Optimality score for the neurologic examination of the infant at 12 and 18 months of age. The Journal of Pediatrics, 135(2), 153–161. 10.1016/s0022-3476(99)70016-8 10431108

[hbm25579-bib-0018] Hadders‐Algra, M. (2014). Early diagnosis and early intervention in cerebral palsy. Frontiers in Neurology, 5(185), 1–13. 10.3389/fneur.2014.00185 25309506 PMC4173665

[hbm25579-bib-0019] Himpens, E., Van den Broeck, C., Oostra, A., Calders, P., & Vanhaesebrouck, P. (2008). Prevalence, type, distribution, and severity of cerebral palsy in relation to gestational age: A meta‐analytic review. Developmental Medicine and Child Neurology, 50(5), 334–340. 10.1111/j.1469-8749.2008.02047.x 18355333

[hbm25579-bib-0020] Hintz, S. R., Barnes, P. D., Bulas, D., Slovis, T. L., Finer, N. N., Wrage, L. A., … Higgins, R. D. (2015). Neuroimaging and neurodevelopmental outcome in extremely preterm infants. Pediatrics, 135(1), e32–e42. 10.1542/peds.2014-0898 25554820 PMC4279063

[hbm25579-bib-0021] Hoon, A. H., Stashinko, E. E., Nagae, L. M., Lin, D. D., Keller, J., Bastian, A., … Johnston, M. V. (2009). Sensory and motor deficits in children with cerebral palsy born preterm correlate with diffusion tensor imaging abnormalities in thalamocortical pathways. Developmental Medicine and Child Neurology, 51(9), 697–704. 10.1111/j.1469-8749.2009.03306.x 19416315 PMC2908264

[hbm25579-bib-0022] Hubermann, L., Boychuck, Z., Shevell, M., & Majnemer, A. (2016). Age at referral of children for initial diagnosis of cerebral palsy and rehabilitation: Current practices. Journal of Child Neurology, 31(3), 364–369. 10.1177/0883073815596610 26239493

[hbm25579-bib-0023] Jensen, E. A., Dysart, K., Gantz, M. G., McDonald, S., Bamat, N. A., Keszler, M., … DeMauro, S. B. (2019). The diagnosis of bronchopulmonary dysplasia in very preterm infants: An evidence‐based approach. American Journal of Respiratory and Critical Care Medicine, 200(6), 751–759. 10.1164/rccm.201812-2348OC 30995069 PMC6775872

[hbm25579-bib-0024] Jeurissen, B., Leemans, A., Jones, D. K., Tournier, J. D., & Sijbers, J. (2011). Probabilistic fiber tracking using the residual bootstrap with constrained spherical deconvolution. Human Brain Mapping, 32(3), 461–479. 10.1002/hbm.21032 21319270 PMC6869960

[hbm25579-bib-0025] Jeurissen, B., Leemans, A., Tournier, J. D., Jones, D. K., & Sijbers, J. (2013). Investigating the prevalence of complex fiber configurations in white matter tissue with diffusion magnetic resonance imaging. Human Brain Mapping, 34(11), 2747–2766. 10.1002/hbm.22099 22611035 PMC6870534

[hbm25579-bib-0026] Jeurissen, B., Tournier, J. D., Dhollander, T., Connelly, A., & Sijbers, J. (2014). Multi‐tissue constrained spherical deconvolution for improved analysis of multi‐shell diffusion MRI data. NeuroImage, 103, 411–426. 10.1016/j.neuroimage.2014.07.061 25109526

[hbm25579-bib-0027] Kaur, S., Powell, S., He, L., Pierson, C. R., & Parikh, N. A. (2014). Reliability and repeatability of quantitative tractography methods for mapping structural white matter connectivity in preterm and term infants at term‐equivalent age. PLoS One, 9(1), e85807. 10.1371/journal.pone.0085807 24475054 PMC3901659

[hbm25579-bib-0028] Kidokoro, H., Neil, J. J., & Inder, T. E. (2013). New MR imaging assessment tool to define brain abnormalities in very preterm infants at term. AJNR. American Journal of Neuroradiology, 34(11), 2208–2214. 10.3174/ajnr.A3521 23620070 PMC4163698

[hbm25579-bib-0029] Kline, J. E., Illapani, V. S., He, L., Altaye, M., Logan, J. W., & Parikh, N. A. (2020). Early cortical maturation predicts neurodevelopment in very preterm infants. Archives of Disease in Childhood, 105(5), 460–465. 10.1136/archdischild-2019-317466 31704737 PMC7205568

[hbm25579-bib-0030] Kline, J. E., Illapani, V. S., He, L., & Parikh, N. A. (2020). Automated brain morphometric biomarkers from MRI at term predict motor development in very preterm infants. NeuroImage: Clinical, 28, 102475. 10.1016/j.nicl.2020.102475 33395969 PMC7649646

[hbm25579-bib-0031] Leergaard, T. B., White, N. S., De Crespigny, A., Bolstad, I., D'Arceuil, H., Bjaalie, J. G., & Dale, A. M. (2010). Quantitative histological validation of diffusion MRI fiber orientation distributions in the rat brain. PLoS One, 5(1), e8595. 10.1371/journal.pone.0008595 20062822 PMC2802592

[hbm25579-bib-0032] Malhotra, A., Sepehrizadeh, T., Dhollander, T., Wright, D., Castillo‐Melendez, M., Sutherland, A. E., … Miller, S. L. (2019). Advanced MRI analysis to detect white matter brain injury in growth restricted newborn lambs. NeuroImage: Clinical, 24, 101991. 10.1016/j.nicl.2019.101991 31473545 PMC6728876

[hbm25579-bib-0033] Novak, I., Morgan, C., Adde, L., Blackman, J., Boyd, R. N., Brunstrom‐Hernandez, J., … Badawi, N. (2017). Early, accurate diagnosis and early intervention in cerebral palsy: Advances in diagnosis and treatment. JAMA Pediatrics, 171(9), 897–907. 10.1001/jamapediatrics.2017.1689 28715518 PMC9641643

[hbm25579-bib-0034] Pannek, K., George, J. M., Boyd, R. N., Colditz, P. B., Rose, S. E., & Fripp, J. (2020). Brain microstructure and morphology of very preterm‐born infants at term equivalent age: Associations with motor and cognitive outcomes at 1 and 2 years. NeuroImage, 221, 117163. 10.1016/j.neuroimage.2020.117163 32663645

[hbm25579-bib-0035] Pannek, K., Scheck, S. M., Colditz, P. B., Boyd, R. N., & Rose, S. E. (2014). Magnetic resonance diffusion tractography of the preterm infant brain: A systematic review. Developmental Medicine and Child Neurology, 56(2), 113–124. 10.1111/dmcn.12250 24102176

[hbm25579-bib-0036] Parikh, N. A. (2016). Advanced neuroimaging and its role in predicting neurodevelopmental outcomes in very preterm infants. Seminars in Perinatology, 40(8), 530–541. 10.1053/j.semperi.2016.09.005 27863706 PMC5951398

[hbm25579-bib-0037] Parikh, N. A. (2018). Are structural magnetic resonance imaging and general movements assessment sufficient for early, accurate diagnosis of cerebral palsy? JAMA Pediatrics, 172(2), 198. 10.1001/jamapediatrics.2017.4812 29279893

[hbm25579-bib-0038] Parikh, N. A., Harpster, K., He, L., Illapani, V. S., Khalid, F. C., Klebanoff, M. A., … Altaye, M. (2020). Novel diffuse white matter abnormality biomarker at term‐equivalent age enhances prediction of long‐term motor development in very preterm children. Scientific Reports, 10, 15920. 10.1038/s41598-020-72632-0 32985533 PMC7523012

[hbm25579-bib-0039] Parikh, N. A., Hershey, A., & Altaye, M. (2019). Early detection of cerebral palsy using sensorimotor tract biomarkers in very preterm infants. Pediatric Neurology, 98, 53–60. 10.1016/j.pediatrneurol.2019.05.001 31201071 PMC6717543

[hbm25579-bib-0040] Raffelt, D. A., Tournier, J. D., Rose, S., Ridgway, G. R., Henderson, R., Crozier, S., … Connelly, A. (2012). Apparent fibre density: A novel measure for the analysis of diffusion‐weighted magnetic resonance images. NeuroImage, 59(4), 3976–3994. 10.1016/j.neuroimage.2011.10.045 22036682

[hbm25579-bib-0041] Raffelt, D. A., Tournier, J. D., Smith, R. E., Vaughan, D. N., Jackson, G., Ridgway, G. R., & Connelly, A. (2017). Investigating white matter fibre density and morphology using fixel‐based analysis. NeuroImage, 144, 58–73. 10.1016/j.neuroimage.2016.09.029 27639350 PMC5182031

[hbm25579-bib-0042] Reijmer, Y. D., Leemans, A., Heringa, S. M., Wielaard, I., Jeurissen, B., Koek, H. L., & Biessels, G. J. (2012). Improved sensitivity to cerebral White matter abnormalities in Alzheimer's Disease with spherical deconvolution based tractography. PLoS One, 7(8), e44074. 10.1371/journal.pone.0044074 22952880 PMC3432077

[hbm25579-bib-0043] Rojas‐Vite, G., Coronado‐Leija, R., Narvaez‐Delgado, O., Ramírez‐Manzanares, A., Marroquín, J. L., Noguez‐Imm, R., … Concha, L. (2019). Histological validation of per‐bundle water diffusion metrics within a region of fiber crossing following axonal degeneration. NeuroImage, 201, 116013. 10.1016/j.neuroimage.2019.116013 31326575

[hbm25579-bib-0044] Romeo, D. M., Cioni, M., Scoto, M., Mazzone, L., Palermo, F., & Romeo, M. G. (2008). Neuromotor development in infants with cerebral palsy investigated by the Hammersmith Infant Neurological Examination during the first year of age. European Journal of Paediatric Neurology: EJPN, 12(1), 24–31. 10.1016/j.ejpn.2007.05.006 17604195

[hbm25579-bib-0045] Rose, J., Cahill‐Rowley, K., Vassar, R., Yeom, K. W., Stecher, X., Stevenson, D. K., … Barnea‐Goraly, N. (2015). Neonatal brain microstructure correlates of neurodevelopment and gait in preterm children 18‐22 mo of age: An MRI and DTI study. Pediatric Research, 78(6), 700–708. 10.1038/pr.2015.157 26322412

[hbm25579-bib-0046] Rose, J., Mirmiran, M., Butler, E. E., Lin, C. Y., Barnes, P. D., Kermoian, R., & Stevenson, D. K. (2007). Neonatal microstructural development of the internal capsule on diffusion tensor imaging correlates with severity of gait and motor deficits. Developmental Medicine and Child Neurology, 49(10), 745–750. 10.1111/j.1469-8749.2007.00745.x 17880643

[hbm25579-bib-0047] Rose, J., Vassar, R., Cahill‐Rowley, K., Guzman, X. S., Hintz, S. R., Stevenson, D. K., & Barnea‐Goraly, N. (2014). Neonatal physiological correlates of near‐term brain development on MRI and DTI in very‐low‐birth‐weight preterm infants. Neuroimage: Clinical, 5, 169–177. 10.1016/j.nicl.2014.05.013 25068107 PMC4110350

[hbm25579-bib-0048] Scheck, S. M., Boyd, R. N., & Rose, S. E. (2012). New insights into the pathology of white matter tracts in cerebral palsy from diffusion magnetic resonance imaging: A systematic review. Developmental Medicine and Child Neurology, 54(8), 684–696. 10.1111/j.1469-8749.2012.04332.x 22646844

[hbm25579-bib-0049] Schilling, K., Gao, Y., Janve, V., Stepniewska, I., Landman, B. A., & Anderson, A. W. (2017). Can increased spatial resolution solve the crossing fiber problem for diffusion MRI? NMR in Biomedicine, 30(12), 1–29. 10.1002/nbm.3787 PMC5685916 28915311

[hbm25579-bib-0050] Spittle, A. J., Cheong, J., Doyle, L. W., Roberts, G., Lee, K. J., Lim, J., … Anderson, P. J. (2011). Neonatal white matter abnormality predicts childhood motor impairment in very preterm children. Developmental Medicine and Child Neurology, 53(11), 1000–1006. 10.1111/j.1469-8749.2011.04095.x 22014319 PMC4040368

[hbm25579-bib-0051] Spittle, A. J., Orton, J., Anderson, P. J., Boyd, R., & Doyle, L. W. (2015). Early developmental intervention programmes provided post hospital discharge to prevent motor and cognitive impairment in preterm infants. The Cochrane Database of Systematic Reviews, 1(11), CD005495. 10.1002/14651858.CD005495.pub4 PMC8612699 26597166

[hbm25579-bib-0052] Tamm, L., Patel, M., Peugh, J., Kline‐Fath, B. M., & Parikh, N. A. (2020). Early brain abnormalities in infants born very preterm predict under‐reactive temperament. Early Human Development, 144, 104985. 10.1016/j.earlhumdev.2020.104985 32163848 PMC7577074

[hbm25579-bib-0053] Teli, R., Hay, M., Hershey, A., Kumar, M., Yin, H., & Parikh, N. A. (2018). Postnatal microstructural developmental trajectory of corpus callosum subregions and relationship to clinical factors in very preterm infants. Scientific Reports, 8(1), 7550. 10.1038/s41598-018-25245-7 29765059 PMC5954149

[hbm25579-bib-0054] Thompson, D. K., Inder, T. E., Faggian, N., Johnston, L., Warfield, S. K., Anderson, P. J., … Egan, G. F. (2011). Characterization of the corpus callosum in very preterm and full‐term infants utilizing MRI. NeuroImage, 55(2), 479–490. 10.1016/j.neuroimage.2010.12.025 21168519 PMC3035727

[hbm25579-bib-0055] Tournier, J. D., Calamante, F., & Connelly, A. (2007). Robust determination of the fibre orientation distribution in diffusion MRI: Non‐negativity constrained super‐resolved spherical deconvolution. NeuroImage, 35(4), 1459–1472. 10.1016/j.neuroimage.2007.02.016 17379540

[hbm25579-bib-0056] Tournier, J. D., Calamante, F., Gadian, D. G., & Connelly, A. (2004). Direct estimation of the fiber orientation density function from diffusion‐weighted MRI data using spherical deconvolution. NeuroImage, 23(3), 1176–1185. 10.1016/j.neuroimage.2004.07.037 15528117

[hbm25579-bib-0057] Tournier, J. D., Mori, S., & Leemans, A. (2011). Diffusion tensor imaging and beyond. Magnetic Resonance in Medicine, 65(6), 1532–1556. 10.1002/mrm.22924 21469191 PMC3366862

[hbm25579-bib-0058] Tournier, J. D., Smith, R., Raffelt, D., Tabbara, R., Dhollander, T., Pietsch, M., … Connelly, A. (2019). MRtrix3: A fast, flexible and open software framework for medical image processing and visualisation. NeuroImage, 202, 116137. 10.1016/j.neuroimage.2019.116137 31473352

[hbm25579-bib-0059] van Kooij, B. J., Van Pul, C., Benders, M. J., Van Haastert, I. C., De Vries, L. S., & Groenendaal, F. (2011). Fiber tracking at term displays gender differences regarding cognitive and motor outcome at 2 years of age in preterm infants. Pediatric Research, 70(6), 626–632. 10.1203/PDR.0b013e318232a963 21857376

[hbm25579-bib-0060] Van't Hooft, J., van der Lee, J. H., Opmeer, B. C., Aarnoudse‐Moens, C. S., Leenders, A. G., Mol, B. W., & de Haan, T. R. (2015). Predicting developmental outcomes in premature infants by term equivalent MRI: Systematic review and meta‐analysis. Systematic Reviews, 4, 71. 10.1186/s13643-015-0058-7 25982565 PMC4438620

[hbm25579-bib-0061] Vassar, R. L., Barnea‐Goraly, N., & Rose, J. (2014). Identification of neonatal white matter on DTI: Influence of more inclusive thresholds for atlas segmentation. PLoS One, 9(12), e115426. 10.1371/journal.pone.0115426 25506943 PMC4266649

[hbm25579-bib-0062] Vincer, M. J., Allen, A. C., Joseph, K. S., Stinson, D. A., Scott, H., & Wood, E. (2006). Increasing prevalence of cerebral palsy among very preterm infants: A population‐based study. Pediatrics, 118(6), e1621–e1626. 10.1542/peds.2006-1522 17074842

[hbm25579-bib-0063] Vogel, J. P., Chawanpaiboon, S., Moller, A. B., Watananirun, K., Bonet, M., & Lumbiganon, P. (2018). The global epidemiology of preterm birth. Best Practice & Research: Clinical Obstetrics & Gynaecology, 52, 3–12. 10.1016/j.bpobgyn.2018.04.003 29779863

[hbm25579-bib-0064] Volpe, J. J. (2009). The encephalopathy of prematurity—Brain injury and impaired brain development inextricably intertwined. Seminars in Pediatric Neurology, 16(4), 167–178. 10.1016/j.spen.2009.09.005 19945651 PMC2799246

[hbm25579-bib-0065] Wang, Y., Wang, Q., Haldar, J. P., Yeh, F. C., Xie, M., Sun, P., … Song, S. K. (2011). Quantification of increased cellularity during inflammatory demyelination. Brain: A Journal of Neurology, 134(12), 3587–3598. 10.1093/brain/awr307 PMC3235568 22171354

[hbm25579-bib-0066] Williams, J., Lee, K. J., & Anderson, P. J. (2010). Prevalence of motor‐skill impairment in preterm children who do not develop cerebral palsy: A systematic review. Developmental Medicine and Child Neurology, 52(3), 232–237. 10.1111/j.1469-8749.2009.03544.x 20002114

[hbm25579-bib-0067] Yoshida, S., Hayakawa, K., Oishi, K., Mori, S., Kanda, T., Yamori, Y., … Matsushita, H. (2011). Athetotic and spastic cerebral palsy: Anatomic characterization based on diffusion‐tensor imaging. Radiology, 260(2), 511–520. 10.1148/radiol.11101783 21555354

[hbm25579-bib-0068] Yoshida, S., Hayakawa, K., Yamamoto, A., Okano, S., Kanda, T., Yamori, Y., … Hirota, H. (2010). Quantitative diffusion tensor tractography of the motor and sensory tract in children with cerebral palsy. Developmental Medicine and Child Neurology, 52, 935–940. 10.1111/j.1469-8749.2010.03669.x 20412261

[hbm25579-bib-0069] Zhang, H., Schneider, T., Wheeler‐Kingshott, C. A., & Alexander, D. C. (2012). NODDI: Practical in vivo neurite orientation dispersion and density imaging of the human brain. NeuroImage, 61(4), 1000–1016. 10.1016/j.neuroimage.2012.03.072 22484410

