# Supplementary material for: Early micro‐ and macrostructure of sensorimotor tracts and development of cerebral palsy in high risk infants
Source: Hum Brain Mapp. 2021 Jul 29;42(14):4708–21. doi: 10.1002/hbm.25579 (PMC8410533; doi:10.1002/hbm.25579)
Supplement: Supplementary file 1 — AppendixS1: Supporting Information [file HBM-42-4708-s001.docx]

Supplementary Material

Early Micro- and Macrostructure of Sensorimotor Tracts and Development of Cerebral Palsy in High Risk Infants

Rahul Chandwani^1^, Julia E. Kline^1^, Karen Harpster^3,4^, Jean Tkach^5,6,7^, Nehal A. Parikh*^1,2^ for the Cincinnati Infant Neurodevelopment Early Prediction Study (CINEPS) Group

^1^Perinatal Institute, Cincinnati Children’s Hospital Medical Center, Cincinnati, OH

^2^Department of Pediatrics, University of Cincinnati College of Medicine, Cincinnati, OH

^3^Division of Occupational Therapy and Physical Therapy, Cincinnati Children’s Hospital Medical Center, Cincinnati, OH

^4^Department of Rehabilitation, Exercise and Nutrition Sciences, University of Cincinnati College of Allied Health Sciences, Cincinnati, OH

^5^Department of Radiology, Cincinnati Children’s Hospital Medical Center, Cincinnati, OH

^6^Imaging Research Center, Department of Radiology, Cincinnati Children’s Hospital Medical Center, Cincinnati, OH

^7^Department of Radiology, University of Cincinnati College of Medicine, Cincinnati, OH

***Corresponding author:**

Nehal A. Parikh, DO, MS

3333 Burnett Ave, MLC 7009

Cincinnati, OH 45229

513-636-7584

Nehal.Parikh@cchmc.org

Materials and Methods

**2.7 Tract Segmentation**

For the corticospinal tract (CST) shown in Supplementary Fig. S1, we located the cerebral peduncle, a heart-shaped central structure, on an axial view. A seed point was placed covering the purple and blue (superior to inferior) fibers of the cerebral peduncle (Fig. S1A). The first waypoint was placed on the second most inferior slice in which the central sulcus was clearly visible. This ROI covered the purple and blue fibers of the precentral gyrus (Fig. S1C). The second waypoint for the CST was placed by locating slices on which the two hemispheres of the thalamus intersect. The portion of the posterior limb of the internal capsule (PLIC) bordering the thalamus was located, and an ROI was placed covering its purple and blue fibers (Fig. S1B). An exclusion mask was drawn on axial and sagittal views, covering the green (anterior to posterior) fibers extending into the cerebellum and the red (left to right) interhemispheric fibers of the brainstem.

For both the motor and sensory components of the superior thalamic radiations (STRM and STRS, respectively), shown in Supplementary Fig. S2, we located the second most inferior slice in which the thalamus was clearly defined and placed a seed point covering all thalamic fibers on an axial view (Fig. S2A&E). The purple and blue fibers of the precentral and postcentral gyri (located near the central sulcus) were included as waypoints for the motor and sensory components, respectively (Fig. S2B&F). Axial exclusion masks were drawn covering the brainstem at the level of the cerebral peduncles, to eliminate any overlapping CST fibers.

For the posterior thalamic radiations (PTR), shown in Supplementary Fig. S3, a seed point was placed by finding the clearest axial slice where the transition from the blue fibers of the corona radiata to the green fibers of the retrolenticular region of the posterior limb was visible. The ROI (Fig. S3A) was placed on the corresponding point in a coronal view, covering the entire pulvinar region of the thalamus. A waypoint was placed in the occipital lobe by first finding the posterior most sagittal slice with the splenium of the CC clearly visible. On a coronal view, we counted the number of slices between this point and the posterior most slice of the brain. As per our published methods (Parikh et al., 2019), we counted one fourth of this number of slices posteriorly from the splenium to locate the ideal waypoint in the occipital lobe. A rectangular ROI was placed here on a coronal view, covering all green fibers in the occipital lobe (Fig. S3B). An exclusion mask was drawn covering the cingulum bundle, interhemispheric fibers, and cerebellum on an axial and coronal view.

For the CC (Supplementary Fig. S4), a seed point ROI was placed on the exact midsagittal slice, covering only the red fibers of the CC (Fig. S4A). Exclusion masks were used in frontal, parietal, and occipital lobes where necessary to remove extraneous fiber populations. For all tracts, any voxels with partial volume fractions or intermediate fiber directions were included or excluded appropriately, after iteratively examining the completed tract trajectory.


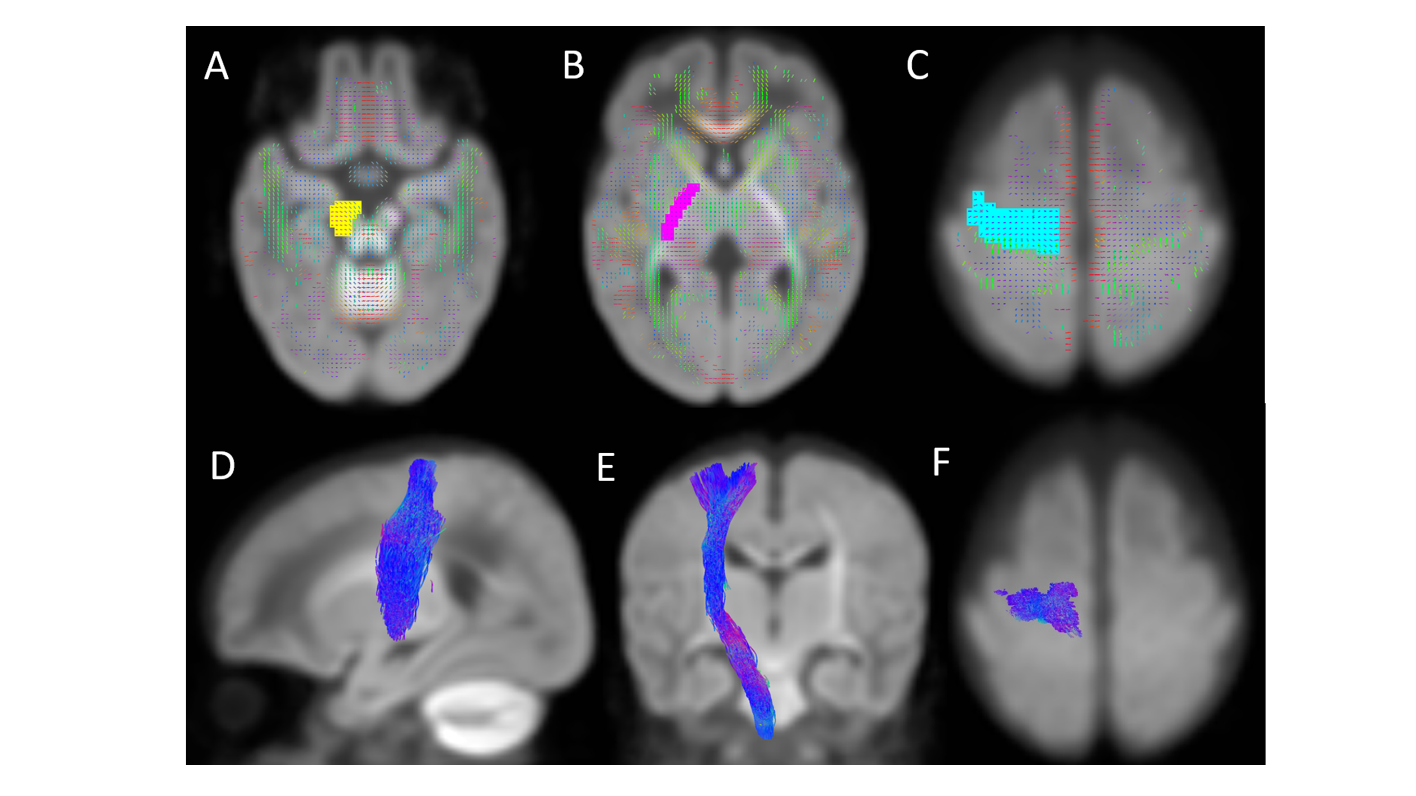


**Figure S1. Location of regions of interest (ROIs) on the group fixel plot and the segmented right corticospinal tract.** (A) Seed point ROI covering the right cerebral peduncle on an axial view; (B) Waypoint ROI covering the right posterior limb of the internal capsule on an axial view; (C) Waypoint ROI covering the right precentral gyrus on an axial view; (D-F) Right corticospinal tract on sagittal, coronal, and axial views, respectively.


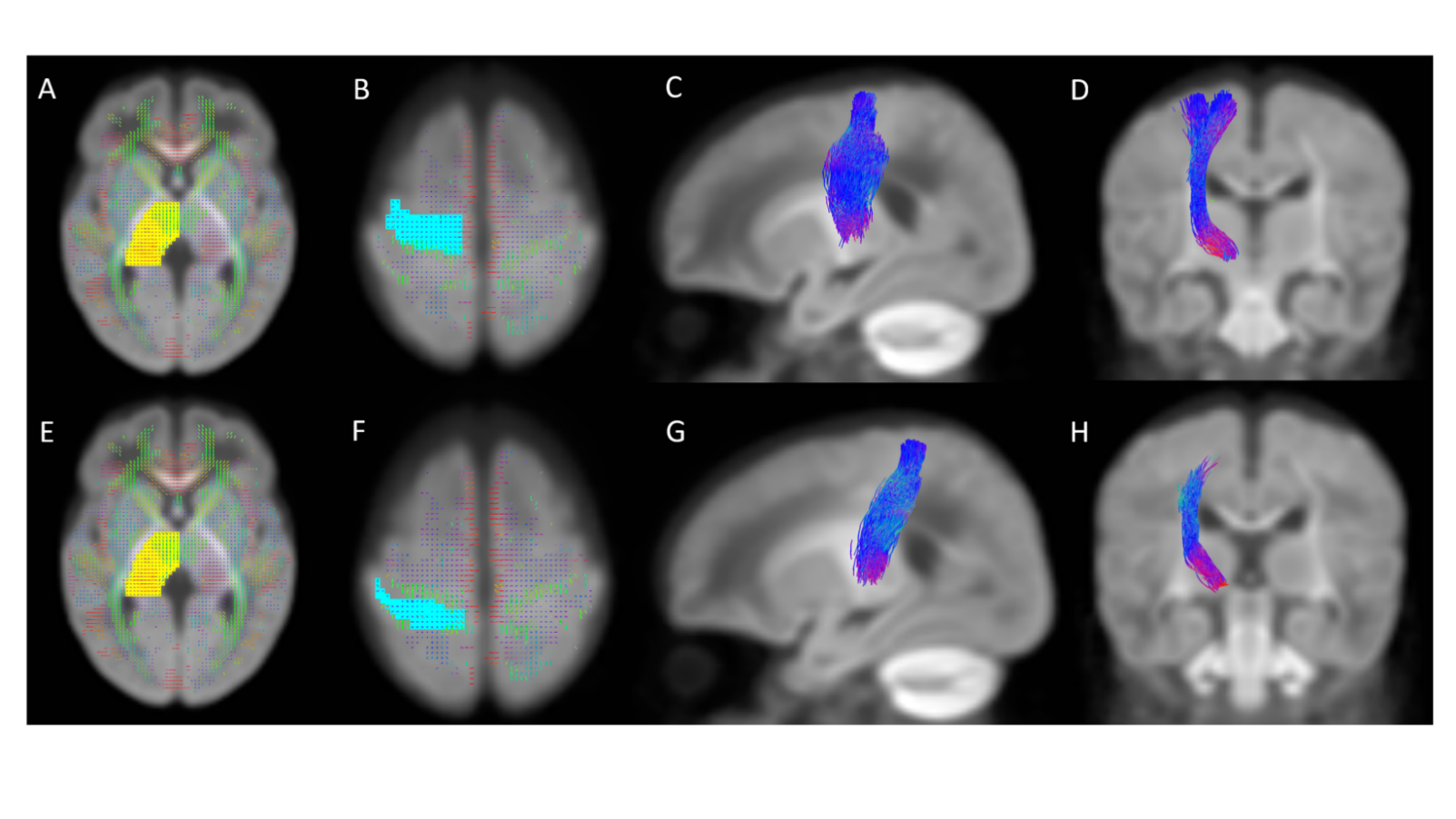


**Figure S2. Location of regions of interest (ROIs) on the group fixel plot and the segmented right superior thalamic radiations (motor and sensory) tracts.** (A,E) Seed point ROI covering the thalamus on an axial view; (B) Waypoint ROI covering the right precentral gyrus on an axial view; (C,D) Right superior thalamic radiations (motor) on sagittal and coronal views, respectively; (F) Waypoint ROI covering the right postcentral gyrus on an axial view; (G, H) Right superior thalamic radiations (sensory) on sagittal and coronal views, respectively.

*All fibers of the STRS are not visible in coronal view, as the tract continues from the thalamus to the post-central gyrus.


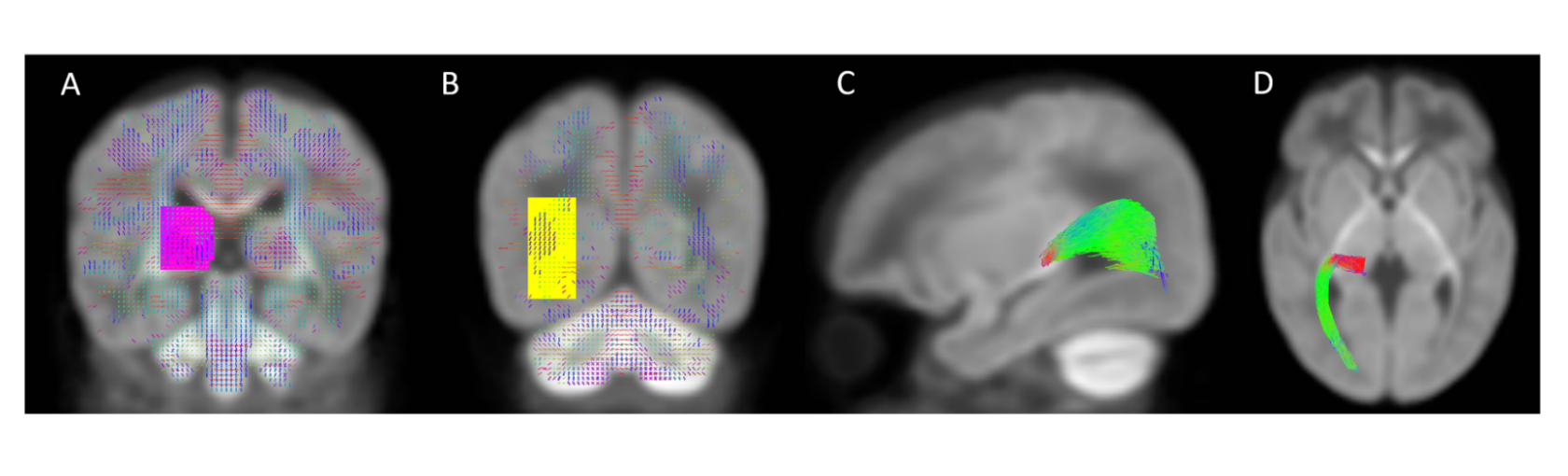


**Figure S3. Location of regions of interest (ROIs) on the fixel plot and the segmented posterior thalamic radiations tract.** (A) Seed point ROI covering the thalamus on a coronal view; (B) Waypoint ROI placed in the occipital lobe on a coronal view; (C,D) Right posterior thalamic radiations on sagittal and axial view, respectively.


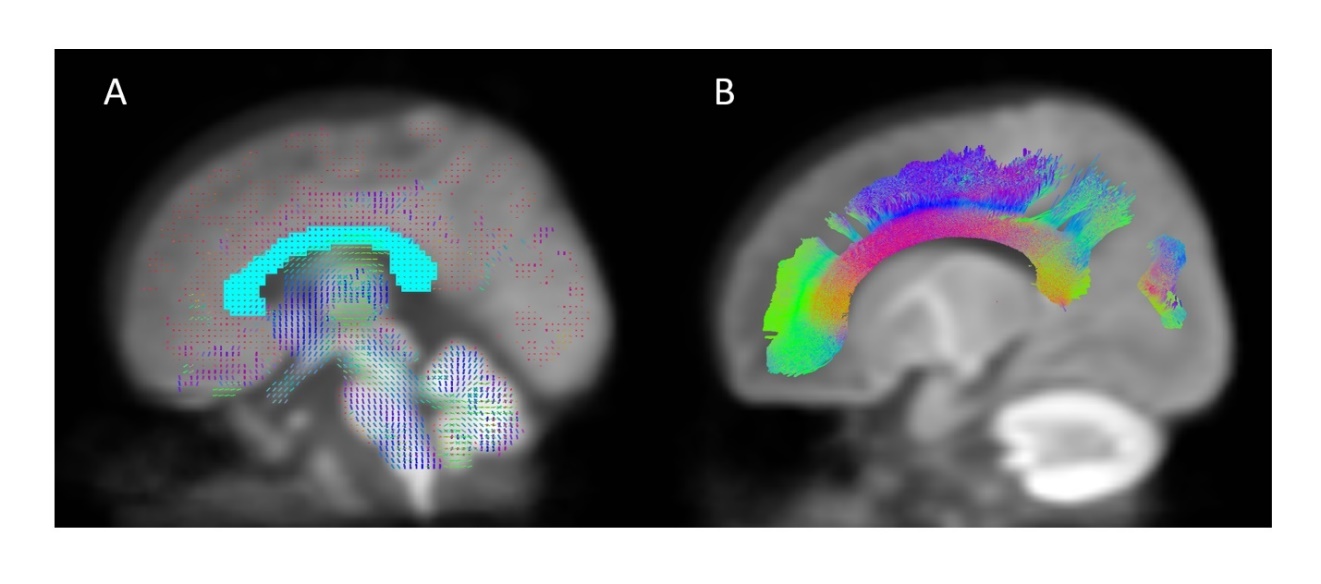


**Figure S4. Location of region of interest (ROI) on the fixel plot and the segmented corpus callosum tract.** (A) Seed point ROI covering the midsagittal corpus callosum on a sagittal view; (B) corpus callosum tract on a sagittal view.

*All fibers of the CC are not visible in sagittal view, as the tract continues from the splenium to the posterior brain.

**Table S1.** Sensorimotor Tract Fiber Density (FD), Fiber Cross-Section (FC), and Fiber Density and Cross-Section (FDC) Differences Between Very Preterm Infants With and Without Early Diagnosis of Cerebral Palsy (CP).

| **Sensorimotor Tract*** | **Low Risk CP**  **(N = 209)** | **Early CP**  **(N = 14)** |  |
| --- | --- | --- | --- |
|  | Mean (SD) | Mean (SD) | P value |
| Corticospinal Tract (CST) |  |  |  |
| FD (R) | 0.449 (0.030) | 0.401 (0.055) | *0.003* |
| FD (L) | 0.447 (0.030) | 0.390 (0.057) | *<0.001* |
| FC (R) | 0.908 (0.101) | 0.793 (0.120) | *<0.001* |
| FC (L) | 0.912 (0.096) | 0.798 (0.118) | *<0.001* |
| FDC (R) | 0.397 (0.070) | 0.292 (0.102) | *<0.001* |
| FDC (L) | 0.398 (0.068) | 0.283 (0.101) | *<0.001* |
| Superior Thalamic Radiations (STR) - motor |  |  |  |
| FD (R) | 0.391 (0.031) | 0.351 (0.049) | *0.003* |
| FD (L) | 0.385 (0.029) | 0.338 (0.053) | *<0.001* |
| FC (R) | 0.900 (0.109) | 0.789 (0.131) | *0.001* |
| FC (L) | 0.903 (0.100) | 0.796 (0.124) | *0.001* |
| FDC (R) | 0.340 (0.072) | 0.247 (0.099) | *<0.001* |
| FDC (L) | 0.337 (0.067) | 0.239 (0.095) | *<0.001* |
| Superior Thalamic Radiations (STR) – sensory |  |  |  |
| FD (R) | 0.387 (0.029) | 0.350 (0.051) | *0.009* |
| FD (L) | 0.372 (0.029) | 0.323 (0.054) | *<0.001* |
| FC (R) | 0.922 (0.092) | 0.804 (0.127) | *<0.001* |
| FC (L) | 0.921 (0.089) | 0.803 (0.115) | *<0.001* |
| FDC (R) | 0.345 (0.063) | 0.256 (0.099) | *<0.001* |
| FDC (L) | 0.333 (0.060) | 0.233 (0.092) | *<0.001* |
| Posterior Thalamic Radiations (PTR) |  |  |  |
| FD (R) | 0.298 (0.032) | 0.258 (0.054) | *0.005* |
| FD (L) | 0.303 (0.317) | 0.258 (0.053) | *0.002* |
| FC (R) | 0.997 (0.082) | 0.884 (0.123) | *<0.001* |
| FC (L) | 1.003 (0.081) | 0.906 (0.116) | *0.003* |
| FDC (R) | 0.289 (0.047) | 0.214 (0.075) | *<0.001* |
| FDC (L) | 0.297 (0.049) | 0.220 (0.077) | *<0.001* |
| Corpus Callosum (CC) |  |  |  |
| FD | 0.288 (0.038) | 0.246 (0.043) | *<0.001* |
| FC | 0.991 (0.087) | 0.901 (0.111) | *0.002* |
| FDC | 0.278 (0.053) | 0.206 (0.065) | *<0.001* |

*All values for fiber density (FD), fiber-bundle cross section (FC), and fiber density and cross-section (FDC) are corrected for postmenstrual age at MRI scan.

**Table S2.** Logistic Regression Analysis between Sensorimotor Tract Fiber Density (FD) and Cerebral Palsy, With and Without Adjustment for Clinical Covariates.

|  | **FB metrics corrected for PMA at MRI only** |  | **FB metrics corrected for PMA at MRI and covariates*** |  |  | **FB metrics corrected for PMA at MRI only** |  | **FB metrics corrected for PMA at MRI and covariates*** |  |
| --- | --- | --- | --- | --- | --- | --- | --- | --- | --- |
| **Tract FD** | Coef.  (95% CI) | P Value | Coef.  (95% CI) | P Value | **Tract FD** | Coef.  (95% CI) | P Value | Coef.  (95% CI) | P Value |
| **RCST** | -30.467  (-44.575,  -16.358) | *<0.001* | -28.844  (-43.361,  -14.328) | *<0.001* | **LCST** | -34.216  (-49.105,  -19.328) | *<0.001* | -32.983  (-48.446,  -17.520) | *<0.001* |
| **RSTRM** | -28.606  (-43.173,  -14.039) | *<0.001* | -27.139  (-42.353,  -11.925) | *<0.001* | **LSTRM** | -33.054  (-48.248,  -17.861) | *<0.001* | -31.814  (-47.869,  -15.760) | *<0.001* |
| **RSTRS** | -29.021  (-44.177,  -13.864) | *<0.001* | -27.746  (-43.784,  -11.708) | *0.001* | **LSTRS** | -34.740  (-50.627,  -18.853) | *<0.001* | -33.753  (-50.617,  -16.889) | *<0.001* |
| **RPTR** | -26.202  (-39.997,  -12.406) | *<0.001* | -22.855  (-37.851,  -7.859) | *0.003* | **LPTR** | -28.774  (-42.789,  -14.759) | *<0.001* | -25.303  (-40.167,  -10.439) | *0.001* |
| **CC** | -23.243  (-36.624,  -9.861) | *0.001* | -19.442  (-33.243,  -5.640) | *0.006* |  | | | | |

Abbreviations: corticospinal tract (Right – RCST, Left – LCST), superior thalamic radiations motor (Right – RSTRM, Left – LSTRM), superior thalamic radiations sensory (Right – RSTRS, Left – LSTRS), posterior thalamic radiations (Right – RPTR, Left – LPTR), corpus callosum (CC), fixel-based (FB), postmenstrual age (PMA)

**Table S3.** Logistic Regression Analysis between Sensorimotor Tract Fiber-Bundle Cross-Section (FC) and Cerebral Palsy, With and Without Adjustment for Clinical Covariates.

|  | **FB metrics corrected for PMA at MRI only** |  | **FB metrics corrected for PMA at MRI and covariates*** |  |  | **FB metrics corrected for PMA at MRI only** |  | **FB metrics corrected for PMA at MRI and covariates*** |  |
| --- | --- | --- | --- | --- | --- | --- | --- | --- | --- |
| **Tract**  **FC** | Coef.  (95% CI) | P Value | Coef.  (95% CI) | P Value | **Tract FC** | Coef.  (95% CI) | P Value | Coef.  (95% CI) | P Value |
| **RCST** | -11.132  (-17.152,  -5.112) | *<0.001* | -9.016  (-15.410,  -2.621) | *0.006* | **LCST** | -11.937  (-18.087,  -5.788) | *<0.001* | -9.879  (-16.389,  -3.369) | *0.003* |
| **RSTRM** | -9.358  (-14.871,  -3.845) | *0.001* | -7.557  (-13.338,  -1.776) | *0.010* | **LSTRM** | -10.420  (-16.284,  -4.557) | *<0.001* | -8.595  (-14.704,  -2.486) | *0.006* |
| **RSTRS** | -12.639  (-18.987,  -6.290) | *<0.001* | -11.137  (-17.905,  -4.369) | *0.001* | **LSTRS** | -13.461  (-19.987,  -6.935) | *<0.001* | -12.017  (-18.810,  -5.224) | *0.001* |
| **RPTR** | -13.677  (-20.333,  -7.021) | *<0.001* | -11.699  (-18.579,  -4.819) | *0.001* | **LPTR** | -12.210  (-18.675,  -5.744) | *<0.001* | -9.946  (-16.686,  -3.207) | *0.004* |
| **CC** | -10.862  (-17.143,  -4.581) | *0.001* | -8.863  (-15.728,  -1.997) | *0.011* |  | | | | |

Abbreviations: corticospinal tract (Right – RCST, Left – LCST), superior thalamic radiations motor (Right – RSTRM, Left – LSTRM), superior thalamic radiations sensory (Right – RSTRS, Left – LSTRS), posterior thalamic radiations (Right – RPTR, Left – LPTR), corpus callosum (CC), fixel-based (FB), postmenstrual age (PMA)

|  | **FB metrics corrected for PMA at MRI only** |  | **FB metrics corrected for PMA at MRI and covariates*** |  |  | **FB metrics corrected for PMA at MRI only** |  | **FB metrics corrected for PMA at MRI and covariates*** |  |
| --- | --- | --- | --- | --- | --- | --- | --- | --- | --- |
| **Tract**  **FDC** | Coef.  (95% CI) | P Value | Coef.  (95% CI) | P Value | **Tract**  **FDC** | Coef.  (95% CI) | P Value | Coef.  (95% CI) | P Value |
| **RCST** | -25.120  (-31.039,  -19.201) | *<0.001* | -21.645  (-27.509,  -15.781) | *<0.001* | **LCST** | -24.619  (-30.694,  -18.544) | *<0.001* | -21.076  (-27.071,  -15.082) | *<0.001* |
| **RSTRM** | -23.769  (-29.795,  -17.742) | *<0.001* | -20.445  (-26.343,  -14.547) | *<0.001* | **LSTRM** | -23.633  (-30.102,  -17.164) | *<0.001* | -19.997  (-26.316,  -13.677) | *<0.001* |
| **RSTRS** | -27.697  (-34.277,  -21.117) | *<0.001* | -23.871  (-30.362,  -17.380) | *<0.001* | **LSTRS** | -28.601  (-35.453,  -21.749) | *<0.001* | -24.564  (-31.331,  -17.798) | *<0.001* |
| **RPTR** | -41.020  (-49.292,  -32.748) | *<0.001* | -36.108  (-44.805,  -27.411) | *<0.001* | **LPTR** | -43.054  (-50.840,  -35.267) | *<0.001* | -38.419  (-46.681,  -30.157) | *<0.001* |
| **CC** | -39.034  (-46.579,  -31.489) | *<0.001* | -35.212  (-43.288,  -27.135) | *<0.001* |  | | | | |

**Table S4.** Linear Regression Analysis between Sensorimotor Tract Fiber Density Cross-Section (FDC) and Global Brain Abnormality Score, With and Without Adjustment for Clinical Covariates.

Abbreviations: corticospinal tract (Right – RCST, Left – LCST), superior thalamic radiations motor (Right – RSTRM, Left – LSTRM), superior thalamic radiations sensory (Right – RSTRS, Left – LSTRS), posterior thalamic radiations (Right – RPTR, Left – LPTR), corpus callosum (CC), fixel-based (FB), postmenstrual age (PMA)

**Table S5.** Linear Regression Analysis between Sensorimotor Tract Fiber Density Cross-Section (FDC) and Hammersmith Infant Neurological Examination (HINE) Score, With and Without Adjustment for Clinical Covariates.

|  | **FB metrics corrected for PMA at MRI only** |  | **FB metrics corrected for PMA at MRI and covariates*** |  |  | **FB metrics corrected for PMA at MRI only** |  | **FB metrics corrected for PMA at MRI and covariates*** |  |
| --- | --- | --- | --- | --- | --- | --- | --- | --- | --- |
| **Tract FDC** | Coef.  (95% CI) | P Value | Coef.  (95% CI) | P Value | **Tract FDC** | Coef.  (95% CI) | P Value | Coef.  (95% CI) | P Value |
| **RCST** | 20.896  (11.645, 30.146) | *<0.001* | 15.410  (6.185, 24.635) | *0.001* | **LCST** | 21.932  (12.554, 31.311) | *<0.001* | 16.426  (7.088, 25.764) | *0.001* |
| **RSTRM** | 20.058  (10.762, 29.355) | *<0.001* | 15.017  (5.832, 24.201) | *0.001* | **LSTRM** | 22.045  (12.238, 31.853) | *<0.001* | 16.765  (7.084, 26.447) | *0.001* |
| **RSTRS** | 23.782  (13.544, 34.020) | *<0.001* | 17.918  (7.730, 28.105) | *0.001* | **LSTRS** | 26.137  (15.544, 36.731) | *<0.001* | 19.930  (9.365, 30.494) | *<0.001* |
| **RPTR** | 38.387  (25.235, 51.538) | *<0.001* | 28.643  (14.732, 42.555) | *<0.001* | **LPTR** | 37.463  (24.639, 50.287) | *<0.001* | 27.666  (14.048, 41.283) | *<0.001* |
| **CC** | 32.244  (19.935, 44.553) | *<0.001* | 21.699  (8.453, 34.944) | *0.001* |  | | | | |

Abbreviations: corticospinal tract (Right – RCST, Left – LCST), superior thalamic radiations motor (Right – RSTRM, Left – LSTRM), superior thalamic radiations sensory (Right – RSTRS, Left – LSTRS), posterior thalamic radiations (Right – RPTR, Left – LPTR), corpus callosum (CC), fixel-based (FB), postmenstrual age (PMA)

**Table S6.** Logistic Regression Analysis between Sensorimotor Tract Fiber Density Cross-Section (FDC) and Prechtl’s General Movements Assessment (GMA), With and Without Adjustment for Clinical Covariates.

|  | **FB metrics corrected for PMA at MRI only** |  | **FB metrics corrected for PMA at MRI and covariates*** |  |  | **FB metrics corrected for PMA at MRI only** |  | **FB metrics corrected for PMA at MRI and covariates*** |  |
| --- | --- | --- | --- | --- | --- | --- | --- | --- | --- |
| **Tract FDC** | Coef.  (95% CI) | P Value | Coef.  (95% CI) | P Value | **Tract FDC** | Coef.  (95% CI) | P Value | Coef.  (95% CI) | P Value |
| **RCST** | -8.733  (-16.985,  -0.481) | *0.038* | -9.245  (-18.018,  -0.471) | *0.039* | **LCST** | -9.314  (-17.354, -1.274) | *0.023* | -10.552  (-19.427,  -1.676) | *0.020* |
| **RSTRM** | -7.315  (-15.896, 1.265) | 0.095 | -7.692  (-16.595, 1.211) | 0.090 | **LSTRM** | -7.847  (-16.560, 0.866) | 0.078 | -9.018  (-18.383,  0.347) | 0.059 |
| **RSTRS** | -10.146  (-19.405, -0.887) | *0.032* | -10.314  (-19.762,  -0.866) | *0.032* | **LSTRS** | -11.620  (-20.675, -2.565) | *0.012* | -13.103  (-22.956,  -3.250) | *0.009* |
| **RPTR** | -14.772  (-25.450, -4.093) | *0.007* | -18.013  (-30.038,  -5.987) | *0.003* | **LPTR** | -12.874  (-23.485, -2.263) | *0.017* | -16.669  (-28.706,  -4.633) | *0.007* |
| **CC** | -13.610  (-24.561, -2.660) | *0.015* | -16.298  (-28.070,  -4.527) | *0.007* |  | | | | |

Abbreviations: corticospinal tract (Right – RCST, Left – LCST), superior thalamic radiations motor (Right – RSTRM, Left – LSTRM), superior thalamic radiations sensory (Right – RSTRS, Left – LSTRS), posterior thalamic radiations (Right – RPTR, Left – LPTR), corpus callosum (CC), fixel-based (FB), postmenstrual age (PMA)

**Table S7**. Intra-rater reliability measurements for each sensorimotor tract.

| **Sensorimotor Tract** | **Intra-class Correlation Coefficient (ICC)** | **Dice Similarity Index** |
| --- | --- | --- |
| **Corticospinal Tract (CST)** |  |  |
| Right | 0.9999 | 0.9847 |
| Left | 0.9996 | 0.9908 |
| **Superior Thalamic Radiations (STR) - motor** |  |  |
| Right | 0.9996 | 0.9436 |
| Left | 0.9998 | 0.9497 |
| **Superior Thalamic Radiations (STR) – sensory** |  |  |
| Right | 0.9999 | 0.9893 |
| Left | 0.9997 | 0.9845 |
| **Posterior Thalamic Radiations (PTR)** |  |  |
| Right | 0.9994 | 0.9638 |
| Left | 0.9974 | 0.9221 |
| **Corpus Callosum (CC)** | 0.9998 | 0.9588 |
